# Chemical Imaging of Carbide Formation and Its Effect on Alcohol Selectivity in Fischer Tropsch Synthesis on Mn-Doped Co/TiO_2_ Pellets

**DOI:** 10.1021/acscatal.4c03195

**Published:** 2024-08-02

**Authors:** Danial Farooq, Matthew E. Potter, Sebastian Stockenhuber, Jay Pritchard, Antonis Vamvakeros, Stephen W. T. Price, Jakub Drnec, Ben Ruchte, James Paterson, Mark Peacock, Andrew M. Beale

**Affiliations:** † Department of Chemistry, University College London, 20 Gordon Street, London WC1H 0AJ, U.K.; ‡ Research Complex at Harwell, Rutherford Appleton Laboratories, Harwell Science and Innovation Campus, Harwell,Didcot OX11 0FA, U.K.; § Finden, Building R71, Harwell Campus, Oxfordshire OX11 0QX, U.K.; ∥ European Synchrotron Radiation Facility, ID 31 Beamline, BP 220, Grenoble CedexF-38043, France; ⊥ IXRF Systems, 10421 Old Manchaca Road, Suite 620, Austin, Texas 78748, United States; # BP, Applied Sciences, Innovation & Engineering, Saltend, Hull HU12 8DS, U.K.

**Keywords:** cobalt, Fischer−Tropsch, XRD-CT, PDF-CT, Mn-dopant, carbide

## Abstract

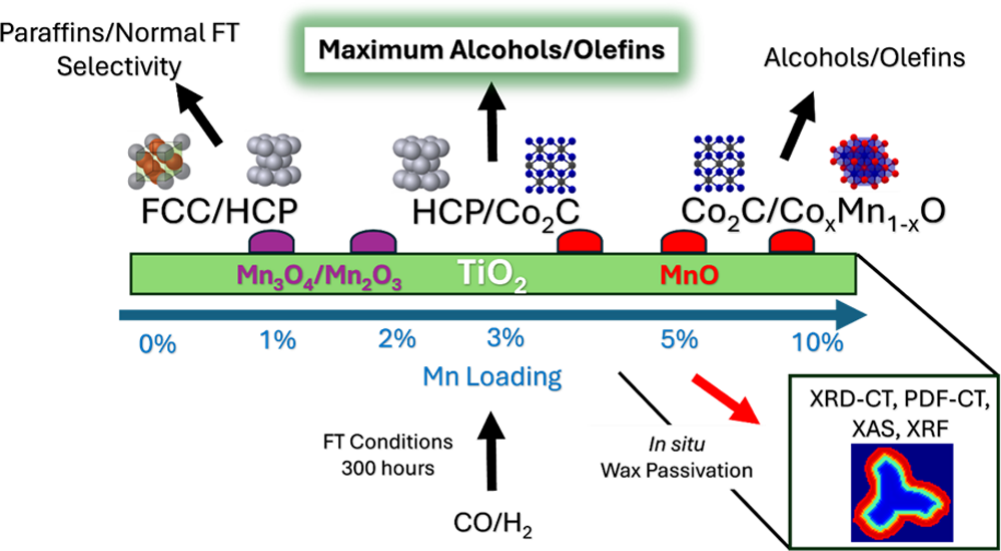

X-ray diffraction/scattering computed tomography (XRS-CT) was used to create two-dimensional images, with 20 μm resolution, of passivated Co/TiO_2_/Mn Fischer–Tropsch catalyst extrudates postreaction after 300 h on stream under industrially relevant conditions. This combination of scattering techniques provided insights into both the spatial variation of the different cobalt phases and the influence that increasing Mn loading has on this. It also demonstrated the presence of a wax coating throughout the extrudate and its capacity to preserve the Co/Mn species in their state in the reactor. Correlating these findings with catalytic performance highlights the crucial phases and active sites within Fischer–Tropsch catalysts required for understanding the tunability of the product distribution between saturated hydrocarbons or oxygenate and olefin products. In particular, a Mn loading of 3 wt % led to an optimum equilibrium between the amount of hexagonal close-packed Co and Co_2_C phases resulting in maximum oxygenate selectivity. XRS-CT revealed Co_2_C to be located on the extrudates’ periphery, while metallic Co phases were more prevalent toward the center, possibly due to a lower [CO] ratio there. Reduction at 450 °C of a 10 wt % Mn sample resulted in MnTiO_3_ formation, which inhibited carbide formation and alcohol selectivity. It is suggested that small MnO particles promote Co carburization by decreasing the CO dissociation barrier, and the Co_2_C phase promotes CO nondissociative adsorption leading to increased oxygenate selectivity. This study highlights the influence of Mn on the catalyst structure and function and the importance of studying catalysts under industrially relevant reaction times.

## Introduction

The transition to a sustainable future relies on the widespread adoption of circular economy principles such as utilizing waste and maximizing resource efficiency which are essential in our quest to achieve net-zero emissions by 2050. Various X to liquid (XTL) technologies have been studied to explore the feasibility of converting alternative carbon sources such as biomass and waste/oil residues to valuable hydrocarbon products.^1^ The Fischer–Tropsch (FT) process, involving the conversion of syngas (mixture of CO and H_2_) into liquid hydrocarbons, is an established process from the mid-1920s.^2^ The process can play a vital role in mitigating the effects of climate change by providing a pathway to carbon-neutral fuels when using renewable hydrogen.^3^ However, it has recently emerged that the selectivity of the process can be tuned to also produce high-value chemical products (olefins and alcohols) from industrial waste streams and captured carbon dioxide (CO_2_) converted to CO.^4,5^

Cobalt, iron, nickel and ruthenium are the most recognized catalytically active elements in the FT process although ruthenium is too expensive for commercial application.^6^ Sasol developed Fischer–Tropsch (FT) processes in Sasolburg (Sasol I–III) utilizing Fe catalysts from 1955 until the 1990s.^7^ Recently, cobalt catalysts have been favored industrially due to their low-methane selectivity and CO_2_ tolerance.^8^ In 1993, Shell adopted cobalt catalysts in a commercial-scale plant in Malaysia.^9^ Shell further advanced its capabilities by establishing the world’s largest Gas-to-Liquids (GTL) plant in Qatar in 2010, known as the Shell GTL Pearl plant, with a capability of producing 140,000 barrels of liquid products per day.^9^ Additionally, BP and Matthey developed FT technology which was successfully deployed in 2002 in a 300 bbl/day demonstration plant in Nikisiki, Alaska.^8^ Furthermore, Fulcrum BioEnergy are a licensee of BP-Matthey CANS catalyst carriers technology^10^ for its Sierra BioFuels plant in Nevada which aims to convert approximately 175,000 million tonnes of municipal solid waste into 42 million liters of renewable FT product per year. The FT CANS technology has also been licensed to Strategic Biofuels for the Louisiana Green Fuels project (LGF) which aims to convert 1 million tonnes of forestry waste feedstock into 120 million liters of renewable diesel per year.^11^

Both the face-centered cubic (FCC) and the hexagonal close-packed (HCP) Co metal phases are known to be active in the FT process. The HCP phase has demonstrated higher activity^12−17^ and it is found that the HCP phase transforms more readily into cobalt carbide (Co_2_C) where they share a similar stacking sequence (ABAB).^18,19^ While the Co_2_C phase was previously thought to be inactive,^20^ recent research has found it to play a vital role in increased alcohol selectivity.^21,22^ Van Ravenhorst et al. conducted an *operando* XRD study and found that metallic FCC cobalt carburized to Co_2_C and no significant deactivation was measured.^21^ Though, it was suggested that a possible decrease in activity due to carbide formation might have been offset by the increased HCP Co phase content. Co_2_C was found to be stable at FT conditions and is formed inversely proportionally to the H_2_/CO ratio and FT reaction temperature.^23^ Co_2_C nanoprisms demonstrated higher selectivity toward lower olefins which was attributed to exposed (101) and (020) facets while the specific active site for higher olefins requires further research.^24^ Zhao et al. investigated model catalytic systems particularly focusing on the complementarity between metallic cobalt and Co_2_C phases, including Co on the surface of Co_2_C nanoparticles and Co_2_C on the surface of Co nanoparticles and found that both exhibited similar alcohol selectivity indicating that the Co–Co_2_C interface present in both systems was the probable active site.^25^ However, Co on Co_2_C led to higher olefin selectivity which was attributed to the lower H_2_/CO syngas ratio. Alkyl chain formation involves both CO dissociation and hydrogenation while alcohol formation involves associative CO adsorption into the alkyl chain which is proposed to occur on the Co–Co_2_C interface.^24,26,27^

A broad range of noble metal promoters have been found to improve FT catalytic performance^28^ and in particular there has been recent interest in the role of Mn promoters in improving selectivity to high value oxygenates such as long-chain alcohols (C_8_–C_20+_).^29^ Mn-promoted FT Co catalysts have demonstrated higher activity, improved selectivity to C_5+_ hydrocarbons and olefins and lower methane production due to the inhibition of hydrogenation activity and a decrease in the CO dissociation barrier.^29,30^ Mn promotes disproportionation and dissociation of CO leading to carburization of Co to Co_2_C. The CO bond dissociation is suggested by Johnson et al. to be promoted by Lewis acid–base interactions between Mn^2+^ ions, situated on the edges of MnO clusters which cover Co particles, and the O atoms of adsorbed CO.^31^ Paterson et al. observed the formation of Co_2_C in post-mortem Co/Mn/TiO_2_ catalysts and it was suggested that the Co metal sites favored chain growth while the MnO/Co_2_C interface facilitated oxygenate selectivity by promoting nondissociative CO insertion.^29^ This is similar to the La dopant on activated carbon which also promotes Co_2_C formation due to the presence of small La oxide clusters in close proximity to Co particles.^32^ Mn promotion was also found to improve cobalt dispersion and reduce the Co particle size.^29,33^ The thermodynamic stability of the Co_2_C phase is improved due to the reduction in Co particle size and increased surface area. Zhu et al. reported that CO dissociation occurs at O vacancies in MnO_*x*_ leading to surface C and carbonate formation.^34^ It is also suggested that Mn inhibits H_2_ uptake on the metallic Co surface further contributing to a lower H_2_/CO ratio promoting Co_2_C formation.^25^ Carbon atoms were thought to diffuse into Co vacancy defects to form the Co_2_C.^25^ Zheng et al. investigated Co/Mn/SiO_2_ catalysts using XRD and observed that the addition of Mn increased the intensity of Co_2_C peaks and led to the formation of Co_3_C (101).^35^

TiO_2_ is found to be a suitable catalyst support material with high surface area, chemical stability and a strong metal support interaction.^36^ Titania supports exhibit a high porosity and pore size, enabling improved dispersion of the active Co metal phase.^37^ TiO_2_ exists in different phases such as anatase, rutile or brookite. The rutile phase has been found to act as a structural promoter enhancing C_5+_ selectivity.^38^ Xaba and de Villiers discovered that the reduction of CoO to Co^0^ required a higher temperature in the anatase and rutile supported catalysts than a P25 catalyst (85% anatase, 15% rutile).^39^ Furthermore, P25 and the rutile supported catalyst were less susceptible to sintering than the anatase catalyst where the P25 catalyst was the most stable. Strong Metal Support Interaction (SMSI) typically occurs in TiO_2_-supported catalyst and is characterized by the encapsulation of metal nanoparticles by a TiO_*x*_ overlayer after high-temperature reduction.^40^ SMSI is reported to be stronger in TiO_2_ catalysts than in those supported by silica, but weaker than in those supported by alumina.^41^ Furthermore, SMSI in TiO_2_-supported Co catalyst have been shown to lead to the blockage of active sites and decreased CO adsorption.^40,42,43^

Due to the multiple complex interactions between different catalytic phases and promoters present it is important to characterize the chemical and physical environments of FT catalyst under industrial conditions to gain a true insight into their performance. Industrial catalysts are often pelletized into millimeter-sized bodies in order to reduce the pressure-drop in the reactor.^44^ Trilobe-shaped extrudates are often used because they offer a better surface area-to-volume ratio and shorter path lengths for the diffusion of reactants and products.^45^ The larger size of these extrudates, compared to powders, leads to spatial variation which require techniques with spatial resolution to comprehend their structure and identify genuine active sites.^46^ Recent advances in X-ray diffraction- and Pair distribution function-computed tomography techniques (XRD-CT and PDF-CT) allow the examination of both crystalline and amorphous phases present in a catalytic body with spatial resolution to gain a better understanding of catalyst structure and function relationships under industrial relevant conditions.^47−49^ Additionally, X-ray fluorescence (XRF) element mapping can be used to provide valuable information about the distribution and concentration of different elements within the catalyst extrudate.^50^ This is crucial for understanding how the catalyst composition changes before and after the reaction.

This study investigates the role of Mn promotion in Co/TiO_2_ Fischer–Tropsch catalysts in increasing alcohol and olefin selectivity using XRD-CT and PDF-CT techniques, performed on recovered catalyst after 300 h of reaction under FT conditions. Conducting these experiments postreaction enabled the exploration of active phases present at more industrially relevant reaction times that usually cannot be reached under time-limited *in situ* beamtime experiments.

## Methods

### Catalytic Reaction

10 wt % Co/TiO_2_ extruded trilobe pellets with varied Mn loadings (0, 1, 2, 3, 5, and 10 wt %) were produced by dissolving cobalt nitrate hexahydrate and manganese acetate tetrahydrate in water, along with P25 titania powder. The catalysts had a fixed 10 wt % loading of Co and increasing Mn loading corresponded with decreasing TiO_2_ content. The Co and Mn solution was impregnated onto the titania and thoroughly mixed. The resulting dry powder was mixed using a Simpson Muller to increase mixing and make a clay like material which was fed to a JMP bonnet Extruder with a single screw, and extruded through 6 JMP extrude dies with 15 trilobe shaped holes with a diameter of 1.6 mm. The extrudates were dried at 120 °C for 24 h and calcined at 300 °C in a box furnace with a ramp rate from room temperature of 20 °C min^–1^. Subsequently, the reduction process was conducted at 300 °C in 100% H_2_ at atmospheric pressure for 15 h before introducing syngas with a temperature ramp of 80 to 150 °C at 2 °C min^–1^ and 150 to 300 °C at 1 °C min^–1^ and dwell at 300 °C. An additional 10% Mn sample was activated at 450 °C (ramp rate of 1 °C min^–1^ from 150 to 450 °C). After cooling to 80 °C at 5 °C min^–1^, 1 g of each catalyst was run under FT conditions for 300 h in 2:1 H_2_:CO syngas with a GHSV of 3000 h^–1^ at 30 barg and 210–240 °C (80 to 190 °C at 0.2 °C min^–1^ and 190 to 205 °C at 0.02 °C min^–1^), where higher Mn containing catalysts required higher temperatures to match conversion. Online GC analysis was used to measure standard metrics such as conversion, short chain selectivity, and productivity as performed in a previous study.^51^ Conversion and selectivity were assessed by comparing the argon internal standard to the input and output of CO using online gas chromatographs. Online measurements covered C_1_ to C_20_, and selectivity was calculated based on the internal standard. The selectivity for C_5_ and higher hydrocarbons (C_5+_) was determined as 100 minus the sum of C_1_–C_4_ products. The reactor was an 8-channel high throughput unit, with common gas feeds and pressures but individual liner temperature control. The catalysts were loaded into each liner ahead of leak testing, activation and FT synthesis. The extrudates were unloaded from the reactor without removing their wax coating (generated *in situ* from the production of long-chain hydrocarbons) as a self-passivating procedure, using only a short nitrogen purge step. A 5% Mn sample was also reacted for 150 h in FT conditions and recovered for analysis, using an analogous 4-channel reactor.^52^

### XRD-CT and PDF-CT Measurements

The pellets were mounted in glass capillaries (3 mm diameter and 0.1 mm wall thickness) and secured with quartz wool. The μ-XRD-CT and μ-PDF-CT scans of the extrudates were performed at ESRF, ID31. A picture of the experimental setup is presented in Figure S1. A monochromatic pencil X-ray beam at 91 keV with a size 5 × 22 μm was used with a 50 ms acquisition time and a PILATUS CdTe 2 M detector. A motorized stage was used to perform the tomographic scans with 120 translation steps (20 μm step size) over 180° (1.5° step size) utilizing an interlaced approach.^53^ The detector was moved from 0.370 to 1.87 m to collect both PDF and XRD data, respectively, for each cross-section of each sample. The XRD images were calibrated using a CeO_2_ standard reference, which was also used to model instrumental broadening of the diffraction patterns.

### Tomographic Reconstruction

After calibration of every 2D diffraction image, pyFAI software^54^ and python scripts were used to azimuthally integrate the images to a 1D powder diffraction pattern.^55^ Air scattering was removed and the sinograms were centered using MATLAB scripts.^56^ The filtered back projection algorithm was used to reconstruct the XRD-CT data. The data was processed into a three-dimensional array (249 × 249 × 924) where the 249 × 249 pixels corresponded to the 2D cross-section image size and the 924 points stored the complete diffraction pattern for each pixel. The resultant spatial resolution of each pixel was approximately 20 μm.

### XRD Refinements

TOPAS Academic v7 was used to perform Rietveld analysis on the XRD-CT data for quantitative phase analysis and structure determination.^57^ The diffraction pattern of a CeO_2_ calibrant was used to calculate the instrument parameters for the TOPAS refinements (peak shape, primary radius and slit radius). The full profile Rietveld analysis was initially performed on the summed diffraction pattern of the entire XRD-CT data to arrive at a suitable starting model for subsequent sequential refinement of the data in each pixel. Scale factors were refined for each phase followed by lattice parameters and then crystallite size parameters. Crystallographic information on the phases refined are available in Table S1. First, the known dominant TiO_2_ polymorphs, rutile and anatase, in the catalyst phase were included and refined in the input file. Next, cobalt metal phases (FCC and HCP) and cobalt carbide were refined. Due to the similar X-ray scattering effects coefficients of Mn and Co, their oxides could not be refined separately. It is known that mixed metal oxide spinels form which could be detected by an expansion in the lattice parameter of the cobalt oxide phases (CoO and Co_3_O_4_) in the refinement since Mn ions are larger than Co ions.^33^

The trilobe cross sections were divided into individual one-pixel thick layers from the outer periphery to the center using a MATLAB script. The script searched the immediately surrounding pixels of each pixel for nonzero values (pixels without diffraction data) to create a mask of the outermost layer of the extrudate. This layer was then subtracted, and a mask was created for the next subsequent layer. This process was repeated until all layers were characterized with a mask. These individual layers were summed, and the corresponding diffraction patterns were analyzed with the Rietveld method using TOPAS to investigate the chemical structure as a function of the distance from the pellet center. 2D spatial maps of the refined parameters were produced.

### Generating the PDF and Real-Space Refinements

The atomic pair distribution function (PDF) was produced by Fourier transforming the XRD-CT data using PDFGetX3 software which also performed various additive and multiplicative corrections, for instance due to finite *Q* range and atomic scattering factors.^58^ The PDFs were calculated to a *Q*_max_ of 25.3 Å^–1^ and to a *r* value of 50 Å with a *Q*_maxinst_ parameter of 25.3 Å^–1^. Initially, the mean XRD pattern was transformed into a mean PDF pattern and analyzed to build a good starting model for the subsequent sequential refinement that would be performed. Real-space Rietveld refinement was performed using TOPAS which minimizes a residual between the experimental PDF and a calculated PDF using a least-squares optimization approach.^57^ Various macros were used to implement PDF real-space refinement on TOPAS.^59,60^ First, peak broadening and dampening due to instrument factors were refined using the CeO_2_ standard which were subsequently fixed for the refinement of the PDF of the sample. Scale, crystallite size, lattice parameters and atomic displacement parameters (*B*_eq_) were refined sequentially for the mean pattern. There is a gradual dissipation of correlated motion as a function of *r* and this was included in the refinement by modeling the atomic displacement parameters using a spherical function.^60^ Screening of additional structures that were potentially present in the sample was carried out using a web-based program, “PDF in the Cloud”.^59^ The program enabled automated structure refinements to be carried out, using CIF files sourced from a crystallographic server. The refinements were carried out on difference PDFs that were obtained from the difference between the experimental data and the known refined support phases, anatase and rutile, and cobalt phases to determine if any unexpected phases were present. Like in the XRD processing, separate layers from the extrudate trilobes were transformed into PDF’s and refined to further study the extrudate structure as a function of distance from the center of the pellets. The refined parameters were then processed in to 2D phase spatial maps using MATLAB.

### Non-Negative Matrix Factorization (NMF)

Due to the large amount of XRD patterns (∼10,000s) present in an XRD-CT data set, automated analysis is required. However, this can lead to diffuse components and phases being missed. Non-Negative Matrix Factorization (NMF) can be used to computationally extract the individual components or phases present in a data set.^61^ NMF was used in this study to ensure the comprehensive identification of all components within the data, preventing the omission of diffuse components and phases that could occur with manual analysis. The Python scikit-learn package was utilized for Non-Negative Matrix Factorization (NMF) analysis, and a custom Python script was developed to generate masks for different components and their corresponding spectra.^62^ Rietveld refinement was performed on the spectra to identify the different phases present in the different components. NMF analysis was applied analogously to the PDF-CT data.

### XAS Experiment

Mn K-edge X-ray Absorption Near Edge Structure (XANES) measurements were collected on the B18 beamline at Diamond Light Source. Data was collected in fluorescence mode on intact pellets using a QEXAFS setup, with an energy resolution of 0.25 eV, with a fast scanning Si(111) crystal monochromator and a Pt mirror. The *ex situ* samples were undiluted, and run in the “activated” and “used” states. Data was collected over 2 h for each sample. Data was analyzed using the Demeter package Athena.^63^

### μ-XRF Experiments

μ-XRF measurements were performed on the 3% Mn samples before and after the reaction where the cross-section and lateral faces were examined by IXRF (Austin, TX, USA). The X-ray fluorescence (XRF) element mapping was collected using IXRF’s Atlas Micro-XRF spectrometer, with a primary polycapillary source (50 W – 50 kV/1000 μA) that leverages a Rh anode target and is capable of a 5-μm spot size.^64^ The source enters the top of the chamber, which allows for the spot to be completely orthogonal with the sample to achieve a true 5-μm spot size. Multiple scans were made of the samples and the focal plane/Z-stage height were set to achieve a 5-μm spot size. The dwell time at each corresponding pixel ranged from 50 to 200 ms and the samples were scanned under vacuum.

## Results and Discussion

### Fischer–Tropsch Performance

**Figure 1:**
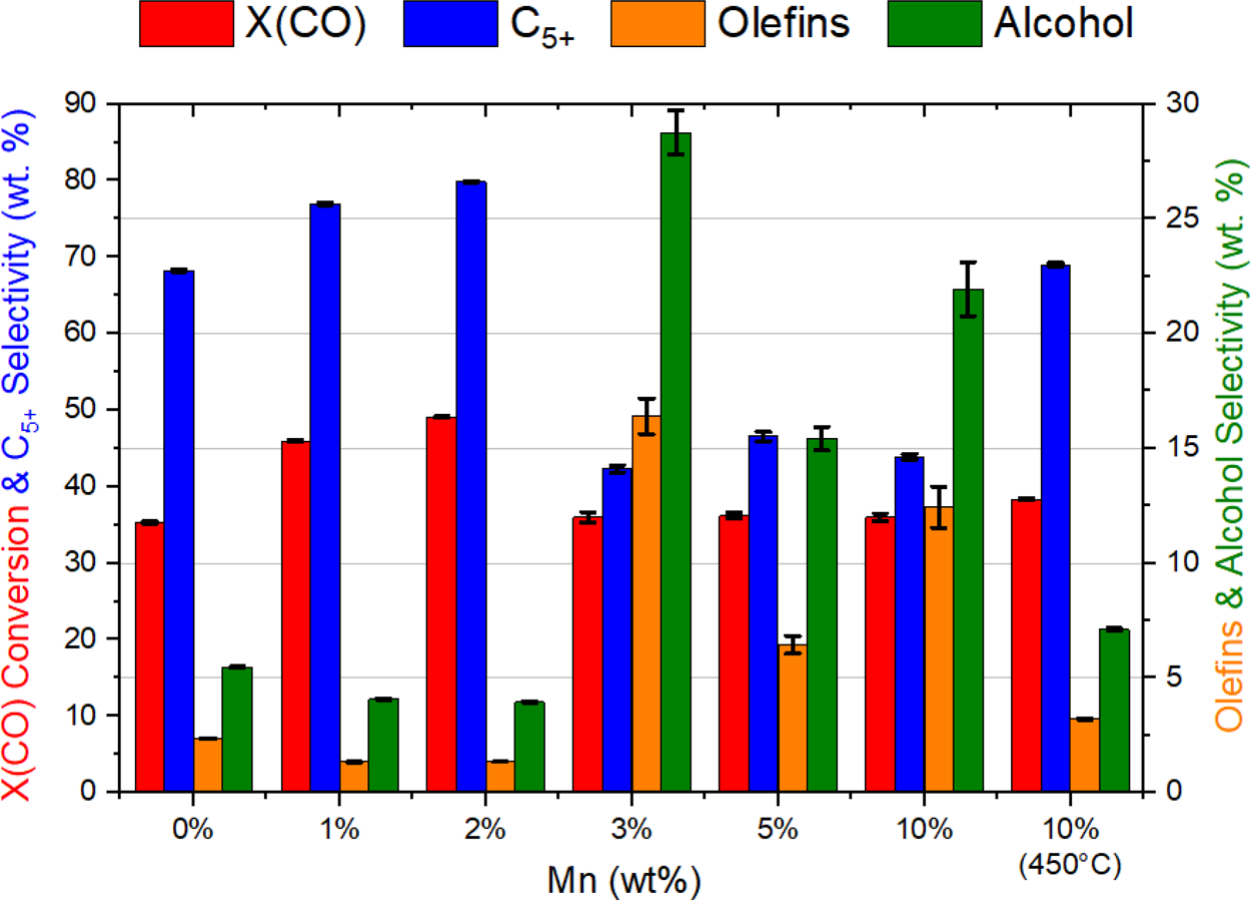
Average CO conversion and C_5+_, alcohol and olefin selectivity (150–270 h) as a function of Mn loading. It was found that an increase in Mn (wt %) results in an increase in C_5+_ selectivity from 0 to 2 wt %, and increased alcohol and olefin selectivity at >3% Mn except for the 10% Mn sample reduced at 450 °C.

Figure 1 presents the average CO conversion and product selectivity for C_5+_ paraffins, olefins, and alcohols for the different Mn samples, calculated over the period from 160 to 270 h. The remaining products in the carbon balance were C_1_–C_4._ The average CO conversion of the 2% Mn sample was the highest (49%) while the 1% Mn sample was marginally lower at 46%. This indicates that the Mn promoter improved conversion, even at low weight percentages, possibly due to smaller crystallite size of the FCC/HCP Co phases. However, at increasing Mn loading (5–10 wt %), conversion was also lower at 36%. The 10% Mn sample which was reduced at 450 °C had an average conversion rate at 38% throughout the experiment which was similar to the 0 wt % sample.

At higher Mn loadings (3–10 wt %), there was an overall increase in alcohol and olefin selectivity compared to the 0–2% Mn samples which had olefin selectivities below 3% and alcohol selectivities below 5%. Specifically, the 3% Mn sample was the optimum loading for selectivity for olefins (16%) and alcohols (29%). Increasing the Mn content beyond 3% led to a decrease in selectivity, with the 5% Mn sample showing lower olefin (6%) and alcohol (15%) selectivities compared to the 10% Mn sample, which had selectivities of 12% and 22% respectively. This suggests that while increasing Mn content from 5% to 10% improved olefin and alcohol selectivity, it did not reach the levels observed with the 3% Mn catalyst.

**Figure 2:**
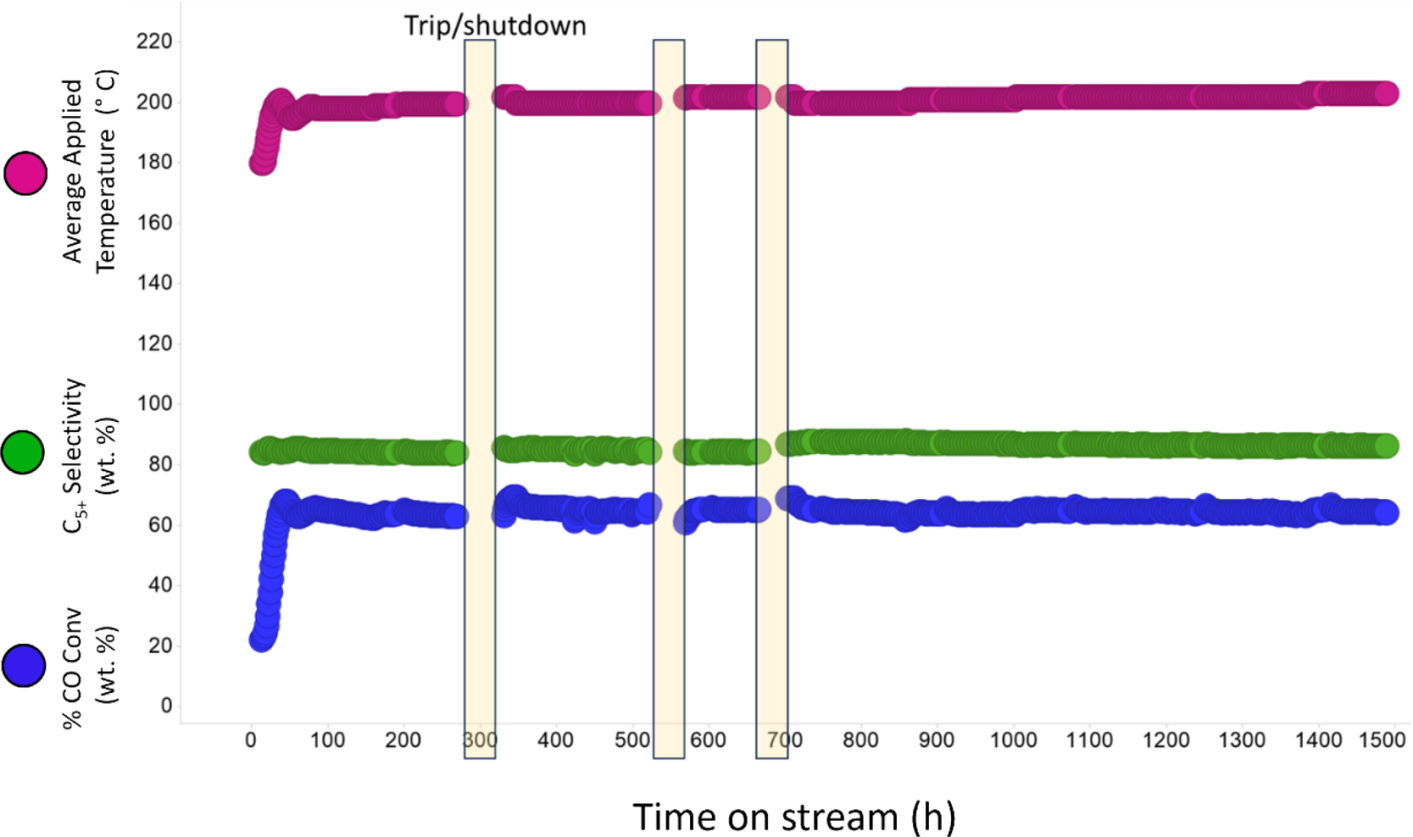
Long-term study of catalyst performance over 1500 h for a 1% Mn sample, revealing the stability of the catalytic pellets. The catalysts had consistent CO conversion and C_5+_ selectivity despite three shutdown events.

C_5+_ selectivity was the highest for the 1 and 2% Mn catalysts (77–80%) suggesting that the Mn promoted chain growth at low weight percentages. The 1 and 2% Mn catalysts exhibited high C_5+_ selectivity and lower olefin and alcohol selectivity. The 10% Mn catalyst which was reduced at 450 °C showed similar selectivity as the 0% Mn catalyst. Catalyst stability was investigated throughout the reaction and the results for the 2% Mn catalyst are presented in Figure 2 where it was found that activity and selectivity was relatively constant from 0–270 h. Furthermore, Figure 2 illustrates that the catalytic activity of a 1% Mn sample remained consistent in a long-term study lasting 1500 h (up to 12 days under FT conditions), in addition to complete recoverability of the catalyst performance after shutdown periods (i.e., loss of power to the unit with no gas or heat supplied). We observe that this immediate resumption of catalytic activity on restarting the reactor suggests no significant evolution in the structure of the catalyst during the intermission.

### Mean XRD-CT Pattern Refinement

**Figure 3:**
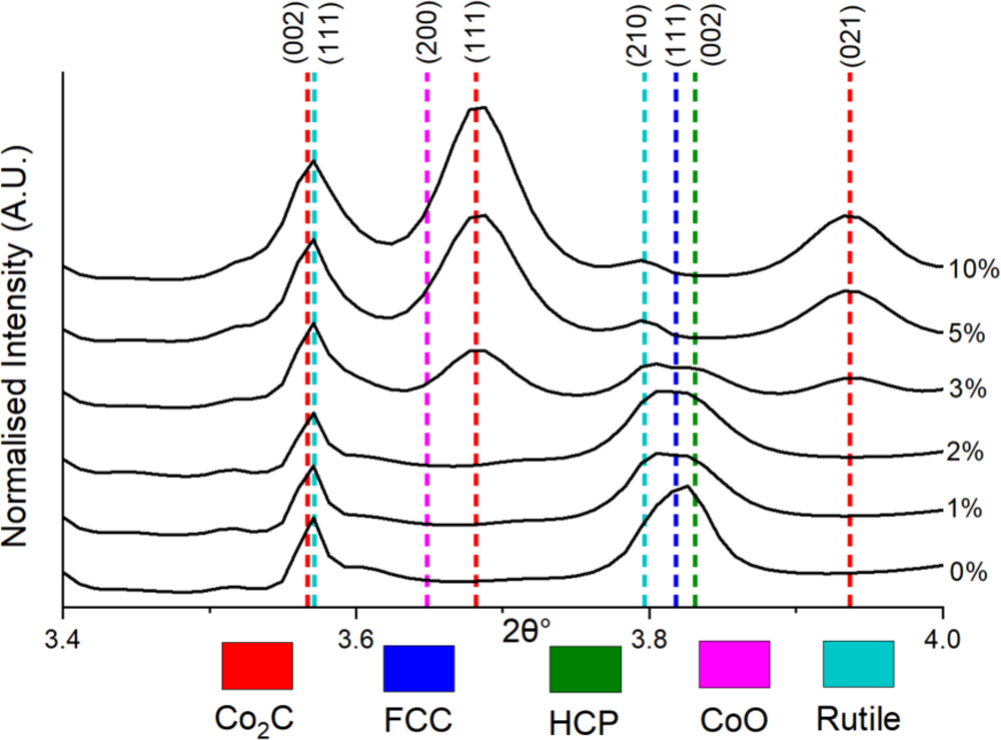
Stacked mean XRD-CT patterns of all the different samples with varied Mn loading (labeled on right) showing the increasing Co_2_C content with increasing Mn loading and the concomitant loss of the Co metal phases (FCC and HCP). Low wt % of CoO was present in the higher Mn loading samples (5 and 10% Mn). Note that no reflections due to the anatase TiO_2_ polymorph are present in this portion of the diffraction pattern.

The mean XRD-CT patterns (illustrated in Figure 3 in the 3.4–4.0 2θ° range) were produced by summing the patterns for all pixels which provided a starting point for analysis. Evidence of the crystalline TiO_2_ support is shown through the (111) rutile peak at 3.56°. It was found that the Co_2_C phase increased (peaks at 3.68° and 3.93°) with Mn loading while the Co^0^ FCC/HCP phases (peak at 3.81°) decreased. This concurs with research from Zheng et al., who also found that increasing Mn loading increased the intensity of Co_2_C peaks.^35^ The presence of Co^0^ metal nanoparticles proved that the wax products remaining on the catalysts prevented oxidation as it is known that Co^0^ nanoparticles oxidize to CoO in ambient conditions.^65^ There was, however, a small percentage (3 wt %) of CoO detected in the 10% Mn sample with an expanded lattice parameter (4.29 Å). A fraction of this could be MnO given that MnO was not separately refined due to the similar scattering factors of Co and Mn. Previous research has also pointed toward the presence of mixed-oxide spinels with an expanded lattice parameter (Co_1–*x*_Mn_*x*_O).^29,66^ Consistent with previous work, no other crystalline Mn-containing species were detected.^29^ The full pattern is presented in Figure S7, where the anatase support phase (TiO_2_) was also identified while MnTiO_3_ was formed in the 10% Mn sample reduced at 450 °C. The anatase and rutile support phases showed little change with varying Mn loading. The mean pattern in Figure S8 highlights the presence of the different Co phases in the 3% Mn sample which contains the Co metal phases (FCC and HCP) and the Co_2_C phase as well as the difference between the refined and experimental data. The FCC (200) peak was excluded from the refinements as it was found to have reduced intensity due to the likely presence of stacking faults.^67^ While this aided the fit of the FCC (100) peak, the presence of stacking faults results in more complex peak shapes and asymmetry that would require more elaborate modeling.^67^

A summary of the Rietveld refinement results is presented in Table 1 (complete results in Table S2). For the 0–2% Mn samples, less than 1 wt % of the Co_2_C phase was present, which correlated with significantly lower olefin and alcohol selectivities compared to the 3–10% Mn catalysts. However, with Mn loading above 3%, the wt % of Co_2_C increased markedly: from 3.6 wt % (3% Mn) to 8.9 wt % (5% Mn) and 7.1 wt % (10% Mn). The lower quantity of Co_2_C in the 3% Mn catalyst was associated with the highest alcohol and olefin selectivities. Conversely, the 5% Mn catalyst, which had the highest Co_2_C content at 8.9 wt %, exhibited lower olefin and alcohol selectivities than the 3% and 10% Mn catalysts, suggesting that excessive Co_2_C hindered these selectivities. Increasing Mn loading from 5% to 10% resulted in reduced Co_2_C formation, potentially due to the increased Mn content blocking Co sites and the formation of the mixed oxide spinels (Co_*x*_Mn_1–*x*_O) inhibiting carburization.

**Table 1:** Rietveld Refinement Results of the XRD-CT Mean Patterns Illustrating the Co Phase wt % and CS (Crystallite Size in nm) in the Samples Extracted after 150 and 300 h^b^

		Mn wt %							
**time (h**		**150**	300						
**phase**		**5%**	**0%**	**1%**	**2%**	**3%**	**5%**	**10%**	**10%**^a^
Co_2_C	wt %	7.5		0.6	0.9	3.6	8.9	7.1	
	CS	7.5				10.6	9.3	11.7	
FCC	wt %	0.4	3.5	3.3	3.6	1.6	0.1	0.0	2.9
	CS	3.54	10.5	8.1	7.7	5.2			11.2
HCP	wt %	0.2	4.9	4.8	3.8	3.1	0.2	0.7	5.3
	CS		2.6	2.2	2.2	7.1		9.9	2.9

a Reduction at 450 °C.

b The Co_2_C wt % increases with Mn loading whilst the Co metal phases (FCC and HCP) decrease with increasing Mn wt % beyond 5%. The 10 wt % samples were reduced at 450 °C.

The crystallite size of the Co FCC phase was found to decrease with increasing Mn loading which correlates with previous research by Paterson et al.^68^ This smaller crystallite size in the 1% and 2% Mn samples was a likely cause for the increased activity and C_5+_ selectivity in these samples. The total Co metal (FCC + HCP + Co_2_C) wt % was found to be approximately 8 wt % (lower than the known 10 wt %) indicating that some of the Co was not found in the XRD data due to small crystallite sizes or disordered Co being present. The low wt % (0.5–2%) of CoO present and the retention of Co_2_C phase affirm the efficacy of the catalyst wax passivation method.

A 5% Mn sample was extracted after 150 h of reaction, and it was found that the Co_2_C phase had already formed at this intermediate stage. The refined weight percentage of the Co_2_C phase increased from 7.5 wt % at 150 h to 8.9 wt % at 300 h and the crystallite size increased from 7.5 to 9.3 nm. The data indicates that carbide had already become the dominant Co phase by 150 h with only marginal further increase observed until 300 h for the samples. This data only shows the bulk average analysis across the samples, akin to the analysis from a laboratory X-ray diffractometer study, where an increase in overall Co_2_C was found with increasing Mn loading. Using this data in combination with XRD-CT analysis enables not just a bulk analysis to be performed but also spatially resolved phase and particle size correlation.

### XRD-CT Refinement

**Figure 4:**
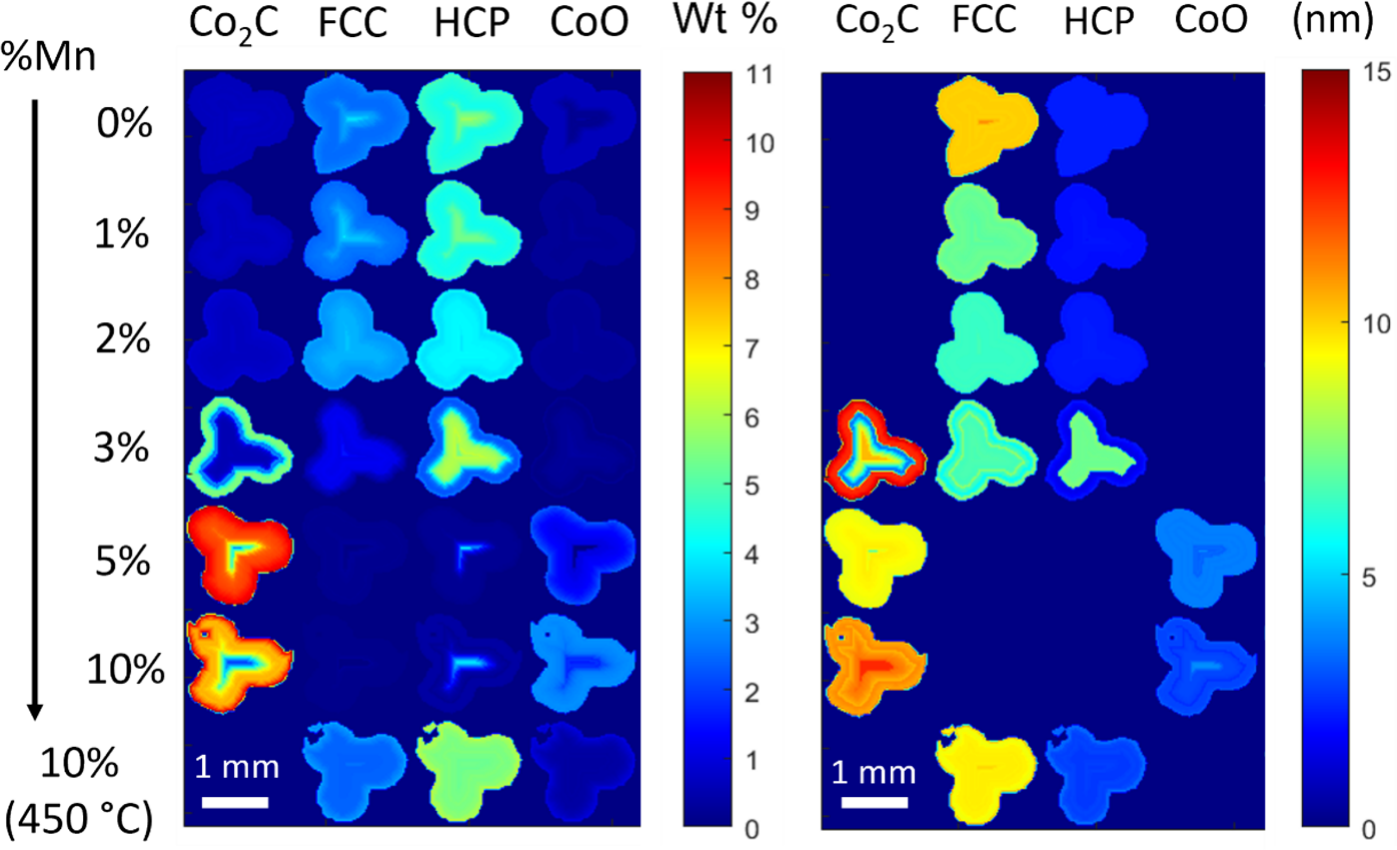
Reconstructed XRD-CT 2D images of the catalytic pellets (extracted after 300 h) illustrating the refined wt % percentage (left) and crystallite size (right) of the different phases present. Increased Mn loading resulted in more cobalt carbide and a smaller particle size of the FCC phase. Lower Mn loadings had a higher presence of FCC and HCP phases. An egg-shell distribution is observed with Co_2_C egg-white and HCP in the center.

**Figure 5:**
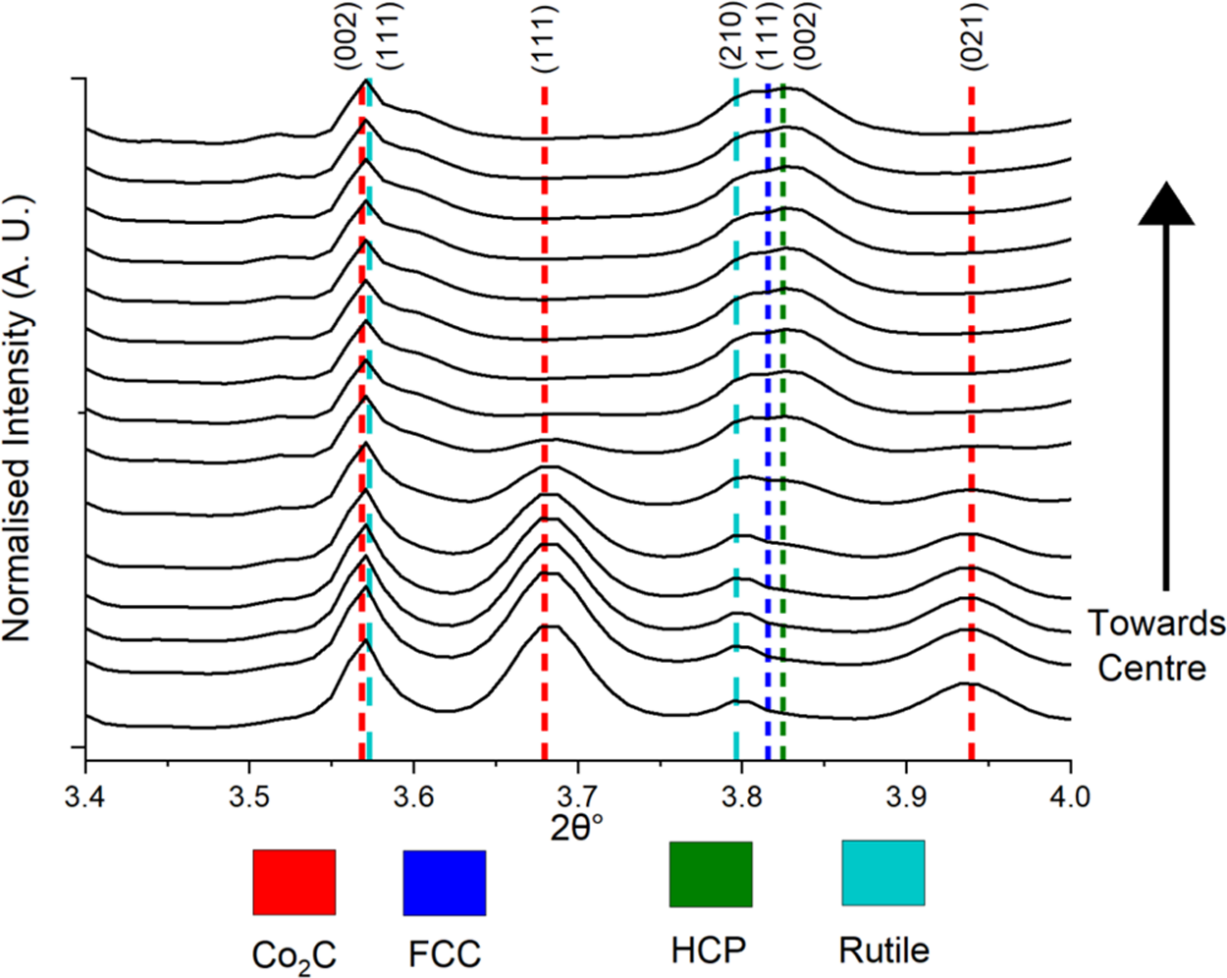
Stacked XRD patterns of the successive layers of the 3% Mn catalyst extrudate showing the evolution of the different cobalt phases with increasing distances (20 μm steps) from the center of the catalyst pellet. Co_2_C is present at the surface periphery while FCC and HCP phases are present at the center.

The XRD-CT spatial mappings presented in Figure 4 illustrate the weight percentages of the different phases present in the catalyst and their crystallite size with spatial resolution. At lower Mn loadings, Co metal (in both FCC and HCP phases) is uniformly distributed, while at higher loadings, it tends to concentrate toward the center of the extrudates. This spatial distribution is further elucidated in Figure 5, which displays XRD patterns of consecutive layers within the 3% Mn catalyst. Here, the Co_2_C phase predominates at the periphery of the extrudates, while FCC and HCP Co metal phases are concentrated at the core. The 3% Mn sample exhibits the highest concentration of the Co HCP phase (7–8 wt %) compared to the 0–2% Mn samples (3–4 wt %) at the central region of the extrudates, evident in the XRD patterns of the successive layers in Figure S9. This observation was not captured in the analysis of the mean XRD patterns, where lower HCP content was found for the 3% Mn sample. This highlights the efficacy of the XRD-CT technique in detecting phase distribution heterogeneities, revealing distinctions between bulk and specific regions. Notably, the 3% Mn sample represents the point just before the significant formation of Co_2_C and an increase in the HCP phase. This suggests an equilibrium between cobalt carburization and reduction back to HCP cobalt, favoring the latter during the reduction process due to their structurally similar hexagonal configuration.^19^ Above this equilibrium point in Mn loading, cobalt carbide phase predominates, while below it, the HCP phase prevails. Other studies have also observed the transformation of the Co_2_C phase to the HCP Co.^19,35^ Strikingly, the 3% Mn sample also exhibited the highest alcohol and olefin selectivity, signifying that an optimal equilibrium between the HCP and Co_2_C phases was reached in this sample, contributing to an enhanced selectivity toward oxygenates. Similar to the mean refinements, the 5% Mn sample showed increased Co_2_C formation, which resulted in reduced alcohol and olefin selectivity compared to the 3 and 10% Mn samples. This suggests that higher Mn content inhibited carburization.

FCC and HCP Co phases were both present in the 10% Mn (reduced at 450 °C) sample and there was no presence of carbides, exhibiting a similar distribution of phases as the 0% Mn sample. Both catalysts also exhibited low selectivity for alcohols and olefins. This is due to the Mn being locked away in the Mn titanate phase which prevents its promotion of carburization by enhancing CO bond dissociation which requires the Mn to be close to the Co.^69^ Only a small percentage of CoO was found in the samples indicating that most of the cobalt was reduced however more was found to be present at higher Mn loadings, likely due to the presence of mixed oxide spinels (Co_1–*x*_Mn_*x*_O). The *R*_wp_ (residual weight percentage) was relatively constant at around 7–8% indicating a good fit for all the patterns.

Like in the mean refinements, it was found that the FCC crystallite size decreases with increasing Mn loading which corresponds to previous research.^29^ The crystallite sizes of the HCP phase (2.5 nm) were much smaller than the FCC phase (6–10 nm) except for the 3% Mn catalyst where larger HCP particles were found to be formed at the center of the extrudates. Larger Co_2_C particles (14 nm) were found to be formed on the periphery of the 3% Mn catalyst compared to the 5 and 10% Mn catalysts (10 nm). This further supports the hypothesis of a Co_2_C to HCP phase transformation equilibrium. This also agrees with previous research that found that Mn in close proximity to larger cobalt particles would carburize.^70^ Small CoO crystallites were found to be present at higher Mn loadings (5 and 10 wt %), which were expected to be mixed-oxide spinels (Co_1–*x*_Mn_*x*_O), indicating that the Mn inhibited reduction of Co, possibly due to SMSI. The phase distribution and crystallite sizes did not vary significantly along the length of the pellet and XRD-CT cross-section maps of the samples at alternative positions within the pellets are presented in Figure S11.

**Figure 6:**
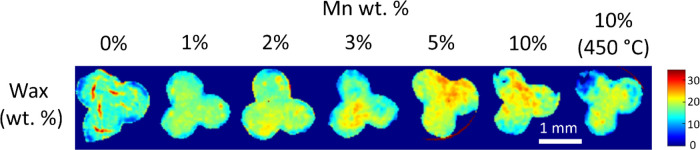
XRD-CT maps of the refined wax (C_3_H_6_)_*n*_ content (wt %) where increasing wt % of wax was found at higher Mn wt %.

Spatial maps of the wax were produced (Figure 6) by refining polyethylene (C_3_H_6_)_*n*_ and it was found that that more wax content remained on the catalyst with increasing Mn loading. This correlates with research that found that olefin products are more likely to readsorb on the catalyst surface, due to greater solubility in synthesis liquids, and undergo secondary reactions such as hydrogenation.^71,72^

### NMF Analysis of XRD-CT Data

NMF analysis was performed to ensure a thorough identification of all phases present in the catalyst. The analysis revealed the presence of five components as further increasing the component count led to the inclusion of components that resembled noise. Due to the homogeneous presence of the support and crystalline wax (C_3_H_6_)_*n*_, all components included these phases. In Figure S12, masked images display the component locations, while Figure S13 illustrate the computed XRD patterns of the components and Table S4 contains the Rietveld refinement results. Component 1 (FCC/HCP phases) was concentrated at the catalyst center with higher Mn loading, while Component 3 (Co_2_C) was primarily located on the catalyst periphery, consistent with the XRD-CT findings. Furthermore, component 2 was found to contain the MnTiO_3_ phase which was found only in the 10% Mn catalyst reduced at 450 °C. Components 4 and 5, demonstrated a colocation of HCP and Co_2_C phases but only accounted 2.4 and 0.3 wt % of the data intensity, respectively. The NMF analysis confirmed that no additional phases were present that were not detected by the XRD-CT analysis.

### PDF-CT Data Analysis

**Figure 7:**
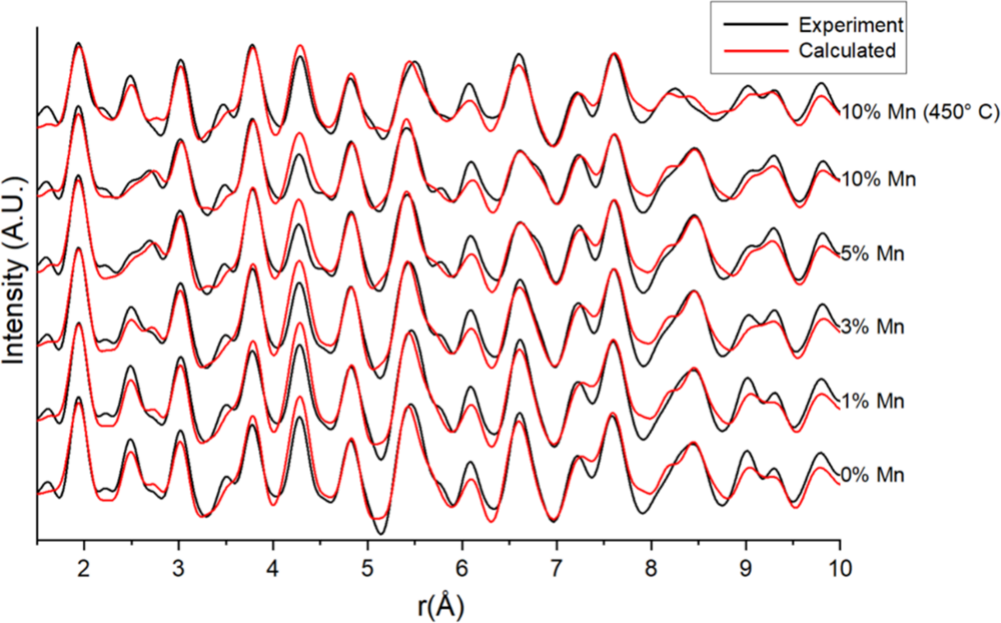
Mean refinements of the PDF data differences. The formation of carbides from 3 to 10% Mn results in the distortion of the FCC/HCP Co–Co peak at 2–3 Å.

The summed PDF patterns were produced by summing all the Bragg data for each pixel and then Fourier transforming the result. Real-space refinement was performed on the patterns and the results are presented in Table S6. The crystallite sizes measured in the PDF analysis were consistently smaller than those obtained from XRD due to smaller, more disordered domains, being detected by PDF.^47^ The refined mean patterns from 0 to 10 Å are presented in Figure 7 where there are changes to the Co–Co peak at 2.5 Å with increasing Mn due to the formation of cobalt carbide. Similar to the XRD refinement, the PDF results indicated increased Co_2_C formation with increasing Mn loading, first appearing at 3% Mn, while Co (FCC and HCP) phases were present at lower Mn loadings (0 and 1 wt %). There was a higher percentage of FCC/HCP cobalt detected in the PDF than in the XRD indicating the presence of small cobalt metal particles. A larger percentage of CoO is detected for the 10% Mn sample indicating that the Mn was present in small mixed-oxide spinels (Co_1_Mn_1–*x*_O) or small MnO particles (1 nm) that could not be detected by XRD. Previous research has also found the presence of small Co particles might inhibit reduction due to SMSI and that small Co particles may also oxidize to CoO.^29,47^

**Figure 8:**
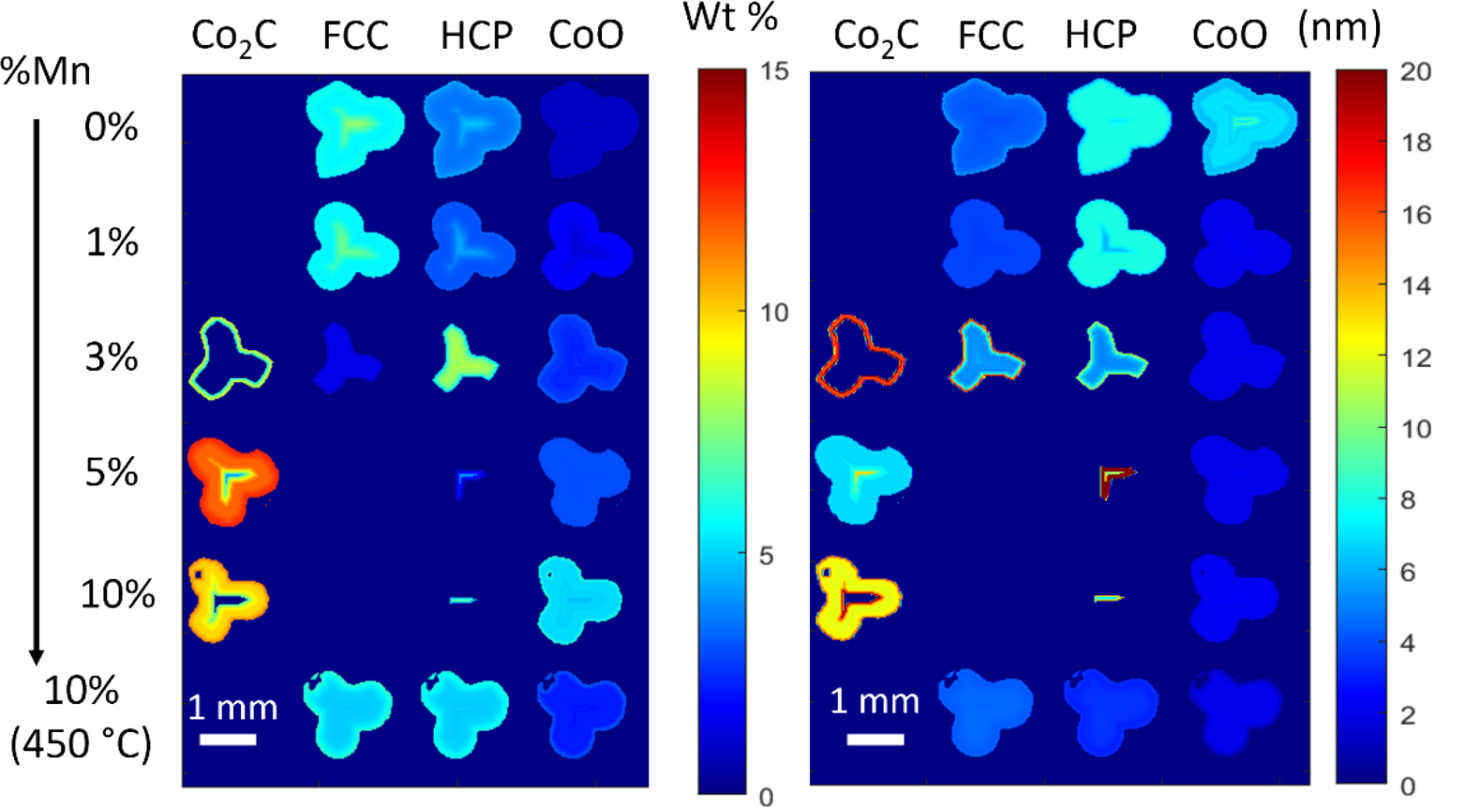
Reconstructed PDF-CT 2D images of the catalytic pellets illustrating the refined wt % percentage (left) and crystallite size (right) of the different phases present in each pixel of the catalyst pellet for the different Mn loadings. Small CoO particles were present at higher Mn loadings while more HCP Co was present at the center of the 3% Mn sample.

The PDF-CT wt % and crystallite size maps are presented in Figure 8. The results from the PDF-CT refinement were consistent with the findings from the XRD-CT refinement where an increasing Mn loading led to an increase in Co_2_C formation on the extrudate periphery and decreasing Co (FCC and HCP) content in the center. Similar to the XRD results, the crystallite size of the FCC particles was found to decrease with increasing Mn loading, correlating with previous research.^29^ A higher HCP wt % was found in the center of the extrudates at higher Mn loadings with the highest concentration present in the 3% Mn sample. This aligns with the XRD results, which revealed a phase equilibrium between the Co_2_C and HCP phases in the 3% Mn sample. The Co_2_C phase was dominant at elevated Mn loading levels, peaking at 5% Mn, and slightly decreasing in the 10% Mn sample.

NMF analysis was also performed on the PDF-CT data to ensure that all phases including noncrystalline phases were detected. The spectra were decomposed into 5 components although only 3 were significant. Two of the components (2 and 4) were of much weaker intensity comprising only 0.1–0.2 wt % of the total intensity. The component locations are shown in Figure S15 and their respectives PDFs are illustrated in Figure S16 while Table S8 contains the Rietveld refinement results. Component 1 contained FCC/HCP Co located at the center of the pellets at higher Mn loading with the presence of small CoO nanoparticles. Component 5 contained the Co_2_C phase that was present on the periphery in the 3–10% Mn samples. Component 3 contained the MnTiO_3_ phase colocated with FCC/HCP Co which was exclusively present in the 10% Mn sample reduced at 450 °C. The NMF analysis confirmed the previous PDF-CT results serving as an efficient tool for spatial decomposition of PDF-CT data into its constituent chemical components.

### XRF Mapping Experiments

μ-XRF images are presented in Figures S17 and S18 which provide a visual insight into the cross sections and lateral faces of the 3% Mn samples both before and after undergoing a 300 h reaction. The images reveal that Mn and Co are colocated in the samples and there were no significant alterations in their distribution observed before and after the extended reaction period, possibly due to SMSI. This opposes recent research that found that Mn oxide and Co were mobile under reaction conditions.^70^

### XAS Analysis

**Figure 9:**
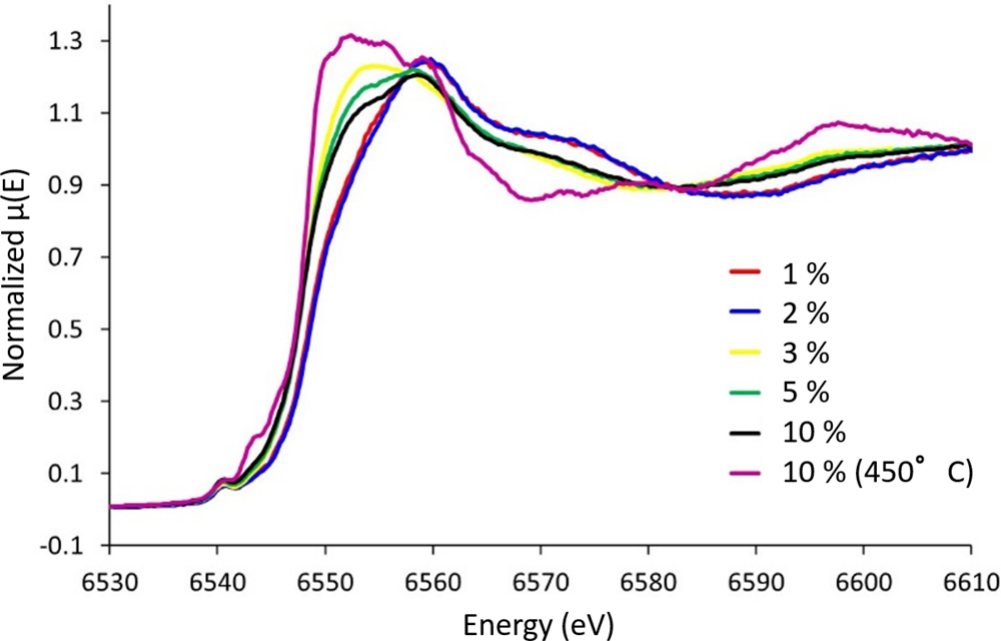
Mn K-edge XANES spectra of CoMn/TiO_2_ species, as a function of Mn weight loading.

To understand how the environment of the Mn species in the catalysts varied with different weight loadings, X-ray Absorption Near Edge Structure (XANES) were collected of the Mn K-edge and compared to a variety of oxidic standards (Figure 9).

All systems shared a common pre-edge feature at 6540 eV, which is attributed to electronic transitions from the 1s to 3d levels. This feature however is known to be largely insensitive to the precise environment compared to the main-edge feature.^73^ Similarly, there is a significant feature at 6560 eV, attributed to dipole-allowed 1s to 4p transitions, which is particularly pronounced in the 1–2% Mn samples.^74^ The XANES data shows excellent agreement with the 1–2% Mn samples (Figure 9, red and blue respectively), suggesting they are in very similar environments. However, as the loading increases, in the main-edge (between 6545 and 6550 eV), there is a notable shift to lower energies, as the Mn weight loading increases, suggesting a reduction in oxidation state. Comparing these spectra with known reference compounds (MnO, MnO_2_, Mn_3_O_4_, Mn_2_O_3_ and MnTiO_3_), Figure S19 shows that the 1–2% Mn systems have a strong resemblance to Mn_3_O_4_ and Mn_2_O_3_ (Figure S19A). This suggests that at least part of the Mn in these catalytic systems are Mn^3+^ in an octahedral environment. The pre-edge peak in the 1 and 2 wt % however is around 1 eV slightly lower than the Mn_3_O_4_ and Mn_2_O_3_ species, this is likely due to a slightly lower average oxidation states in the samples, compared to the oxidic references, or due to their existence as smaller nanoparticles, and not bulk oxides. Above these loadings (>2 wt % Mn, Figure S19B) a signal at 6553 eV begins to evolve, which coincides with the main feature in the reference MnO spectra. The 10% Mn sample reduced at 450 °C presented similar features to the MnTiO_3_ reference, suggesting that postreaction the Mn active sites alloy with the TiO_2_ support, forming a ternary phase. These results have confirmed that Mn remained in an oxidic state, corroborating previous findings that Mn has little impact as a hydrogen spillover enhancer.^68^ Overall, the XANES study suggests that at lower weight loadings (1–2% Mn) the Mn environment resembles Mn_3_O_4_ and Mn_2_O_3_ which correlated with the presence of FCC/HCP Co^0^. At higher weight loadings (3–10% Mn) there were significant features from MnO appearing which existed in conjunction with the Co_2_C phase. The presence of MnO has been suggested to promote CO bond dissociation leading to the carburization of Co to Co_2_C.^31^

## Summary and Conclusions

μ-XRD-CT and μ-PDF-CT (or XRS-CT) techniques were used as complementary techniques to study Co/TiO_2_/Mn FT catalysts recovered after reaction which maintained their state due to *in situ* wax passivation. The protective wax coating, produced from the reaction, prevented the oxidation of the Co nanoparticles which preserved the chemical integrity of the extrudates; we conclude this since ∼5 nm metallic Co NPs were observed in the samples despite their being known to be highly sensitive to oxidation in contact with air/water.^65^ This preservation enabled the X-ray scattering experiments to be conducted after the retrieval of the catalysts following the reaction; we observe the immediate recovery of catalyst performance after multiple reactor shutdowns (Figure 2) which suggests that no significant structural evolution occurs during these intermissions and the performance stabilizes after some ∼50 h of reaction. This approach enabled the study of the state of a real structured and active catalyst under real reaction conditions and operation (300 h), in the absence of being able to *in situ* synchrotron experiments over such time periods and moreover demonstrates the feasibility of this “suspended animation” approach for characterizing spent FT catalysts to understand active states and deactivation phenomena in the future without the need for extensive post reaction treatment.

It was found that increasing Mn loading led to the formation of increasing amounts of MnO which correspondingly led to increasing cobalt carbide content and decreasing cobalt metal (FCC and HCP) in the reacted catalysts. XANES analysis revealed that the Mn was oxidic and increasing MnO was present at higher weight loadings (3–10 wt % Mn) which correlated with increasing olefin and alcohol selectivity. It is thought that the increased Mn leads to a decrease of the CO dissociation barrier promoting carburization of cobalt.^29^ Previous research has found that CO dissociation is more likely to occur on MnO than Mn_3_O_4_.^31,34,75^ XRS-CT revealed that the cobalt carbide formed was located on the periphery of the catalytic extrudates while the cobalt metal phases (FCC and HCP) were at the center. This could be due to a higher partial pressure of CO at the periphery due to pore diffusion limitations of the syngas. Previous research demonstrated that Co_2_C is formed inversely proportionally to the H_2_/CO ratio.^23^ Evidence has also shown that before reduction, aggregated cobalt oxide particles (weak metal–support interactions) which are preferably located at the edges of the extrudates reduce more quickly.^47^ This was suggested due to a higher H_2_ concentration at the center and particle size effects. A sudden increase in alcohol and olefin selectivity at 3% Mn coincided with an increased amount of Co_2_C and the highest HCP concentration indicating that an optimum equilibrium was reached between the two phases. The presence of the two phases contributed synergistically to achieve maximum oxygenate selectivity. Additionally, DFT calculations have found that Co_2_C promotes CO nondissociative adsorption leading to increased oxygenate selectivity.^27^ However, the 5% Mn sample exhibited the highest Co_2_C content and consequently lower selectivity for both alcohols and olefins compared to the 10% Mn sample, possibly due to blockage of Co sites and the formation of mixed metal phases (Co_*x*_Mn_1–*x*_O). This also suggests that the increased Co_2_C content in the 5% Mn sample adversely affected the selectivity of these products. The small Co particles present in the 10% Mn catalyst could have inhibited reduction due to SMSI, as suggested previously.^29^ XRF imaging of the extrudates revealed that there was no detectable change in the sample Co/Mn distribution under reaction conditions which could also be potentially related to SMSI.

The production of Mn titanates (MnTiO_3_), due to reduction at 450 °C during activation, changes the selectivity of the high loading 10% Mn catalyst to that of a 0% Mn catalyst, where there is a higher C_5+_ but lower olefins and alcohol selectivity. The Mn is locked away in Mn titanates and is not able to promote cobalt carbide formation leaving the cobalt metal in the FCC and HCP form.

PDF analysis, which does not assume periodic order, allowed the study of small nanoparticles and disordered phases that were not detectable using XRD. PDF-CT revealed the presence of small CoO particles (∼1 nm) in the 10% Mn sample indicating the presence of mixed oxide spinels (Co_*x*_Mn_1–*x*_O). This suggests that Mn inhibits Co reduction which led to decreased CO conversion and product yield. Higher FCC/HCP Co wt % was found using PDF indicating that a small proportion was present as small nanoparticles that could not be detected in the XRD refinements. Senecal et al. also discovered similar findings with the use of PDF.^47^ NMF analysis was used as an efficient tool to spatially decompose the XRD-CT and PDF-CT data into different chemical components ensuring the detection of all phases.

Recovering and imaging the catalyst after 300 h of reaction provided an insight into the catalytic structure during industrially relevant conditions and time scales. Increasing Co_2_C content was detected with increasing Mn loading which correlated with increasing alcohol and olefin selectivity. Spatial resolution of the phases within the extrudates was provided by using XRD-CT and PDF-CT chemical imaging techniques. Further research is required however to uncover more details regarding the specific role of Mn and potential reaction mechanisms contributing to the increased formation of olefins and oxygenates with the combined presence of Co_2_C and HCP Co.

## Supporting Information Available

Additional information on catalytic results, Rietveld refinement results, XRF images and XAS data (PDF)
