# Supplementary material for: Chemical Imaging of Carbide Formation and Its Effect on Alcohol Selectivity in Fischer Tropsch Synthesis on Mn-Doped Co/TiO2 Pellets
Source: ACS Catal. 2024 Aug 2;14(16):12269–81. doi: 10.1021/acscatal.4c03195 (PMC11334103; doi:10.1021/acscatal.4c03195)
Supplement: Supplementary file 1 — cs4c03195_si_001.pdf [file cs4c03195_si_001.pdf]

Electronic Supplementary Information for:

Chemical imaging of Carbide formation and its effect on Alcohol selectivity in Fischer Tropsch Synthesis on Mn-doped Co/TiO_2 pellets

Danial Farooq[1,2], Matthew Potter[1,2], Sebastian Stockenhuber[1,2], Jay Pritchard[1,2], Antonios Vamvakeros[3], Stephen W. T. Price[3], Jakub Drnec[4], Ben Ruchte[5], James Paterson[6], Mark Peacock[6], Andrew M. Beale[1,2]

[1]*Department of Chemistry, University College London, 20 Gordon Street, WC1H 0AJ, UK*

[2]*Research Complex at Harwell, Rutherford Appleton Laboratories, Harwell Science and Innovation Campus, Harwell, Didcot, OX11 0FA, UK*

[3]*Finden, Building R71, Harwell Campus, Oxfordshire, OX11 0QX, United Kingdom*

[4]*European Synchrotron Radiation Facility, ID 31 Beamline, BP 220, F-38043 Grenoble Cedex, France*

[5]*IXRF Systems, 10421 Old Manchaca Road, Suite 620, Austin, TX 78748, USA*

[6]*BP, Applied Sciences, Innovation & Engineering, Saltend, Hull, UK*

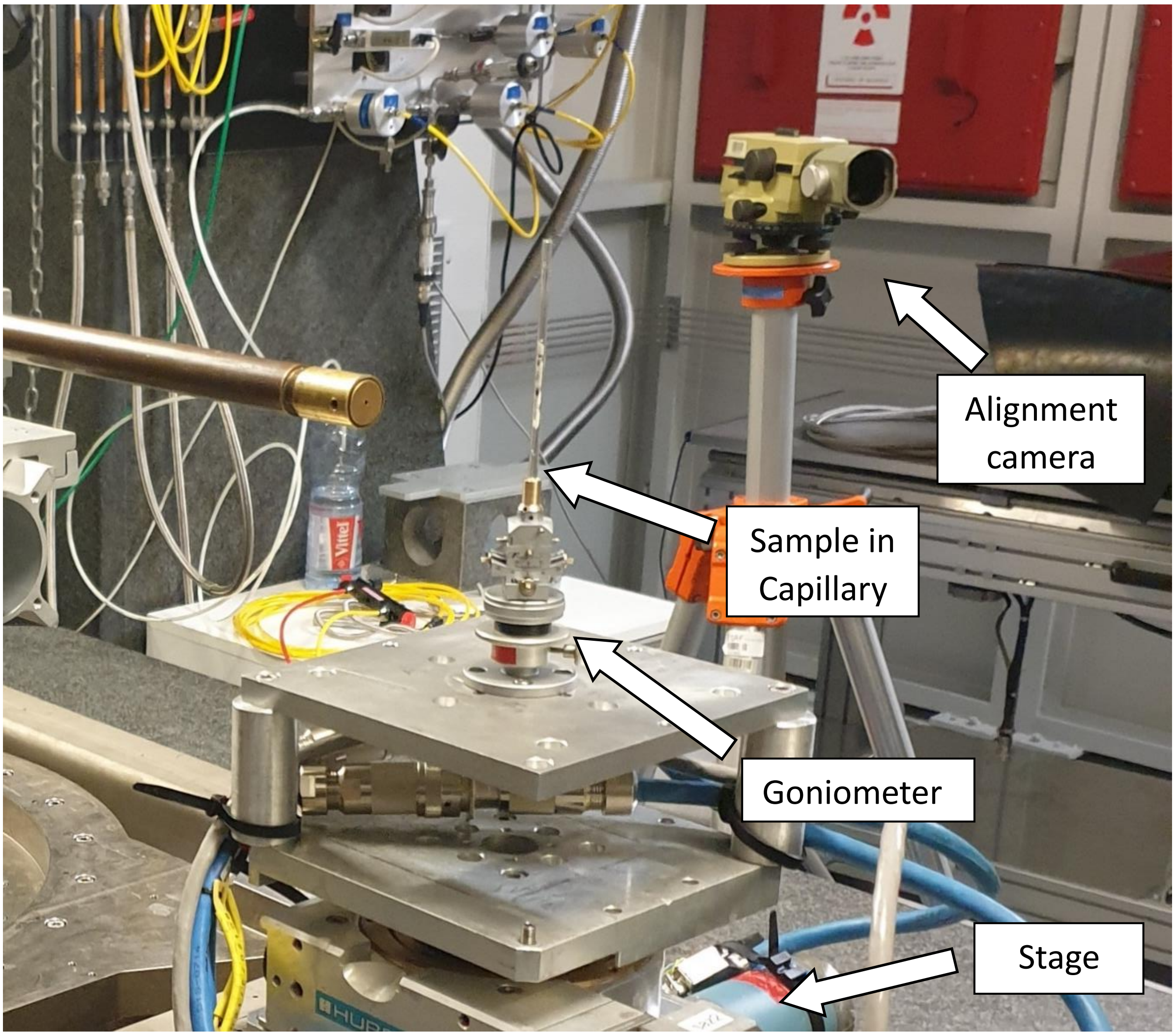

Figure S1 - Experimental set-up at ID31, ESRF, for the XRD-CT and PDF-CT experiments

Table S1 – Crystallographic information of identified phases.

Phase	Space Group	ICSD Code
Co_2C	P m n n (58)	16895
FCC, Co	F m -3 m (225)	41093
HCP, Co	P 63/m m c (194)	44990
CoO	F m -3 m (225)	9865
Rutile, TiO_2	P 42/m n m (136)	9161
Anatase, TiO_2	I 41/ a m d S (141)	9852
$MnTiO_3$	R -3 H (148)	60006

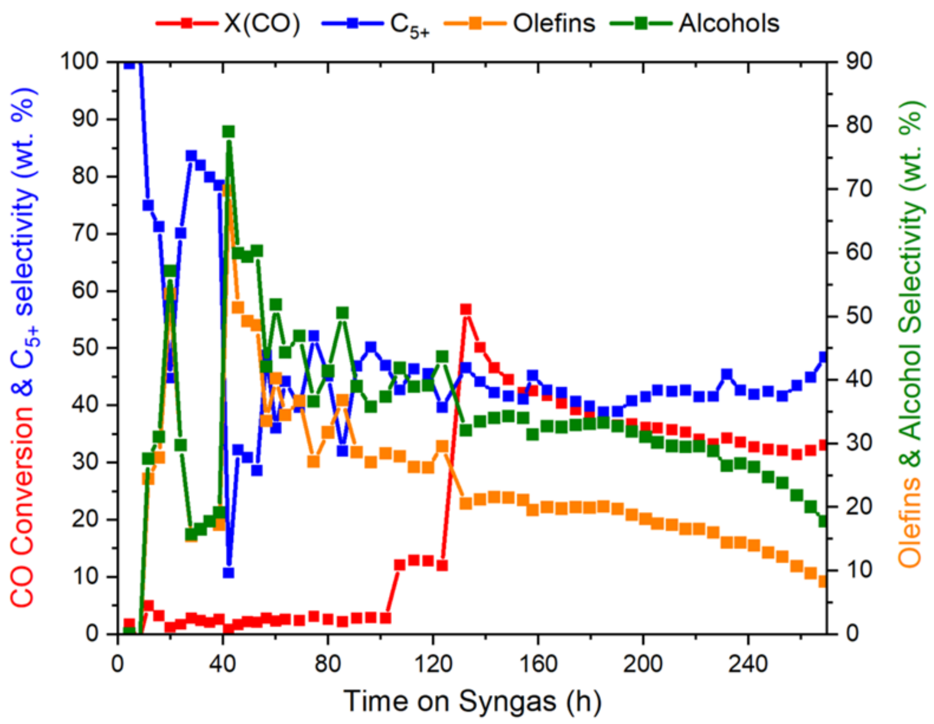

Figure S2 – CO conversion and selectivity to C5+, alcohols and olefins for the 3 % Mn sample for the reaction time from 0 to 270 h at 30 barg and 210-240 °C. The selectivity and conversion are stable with time on stream after 125 h.

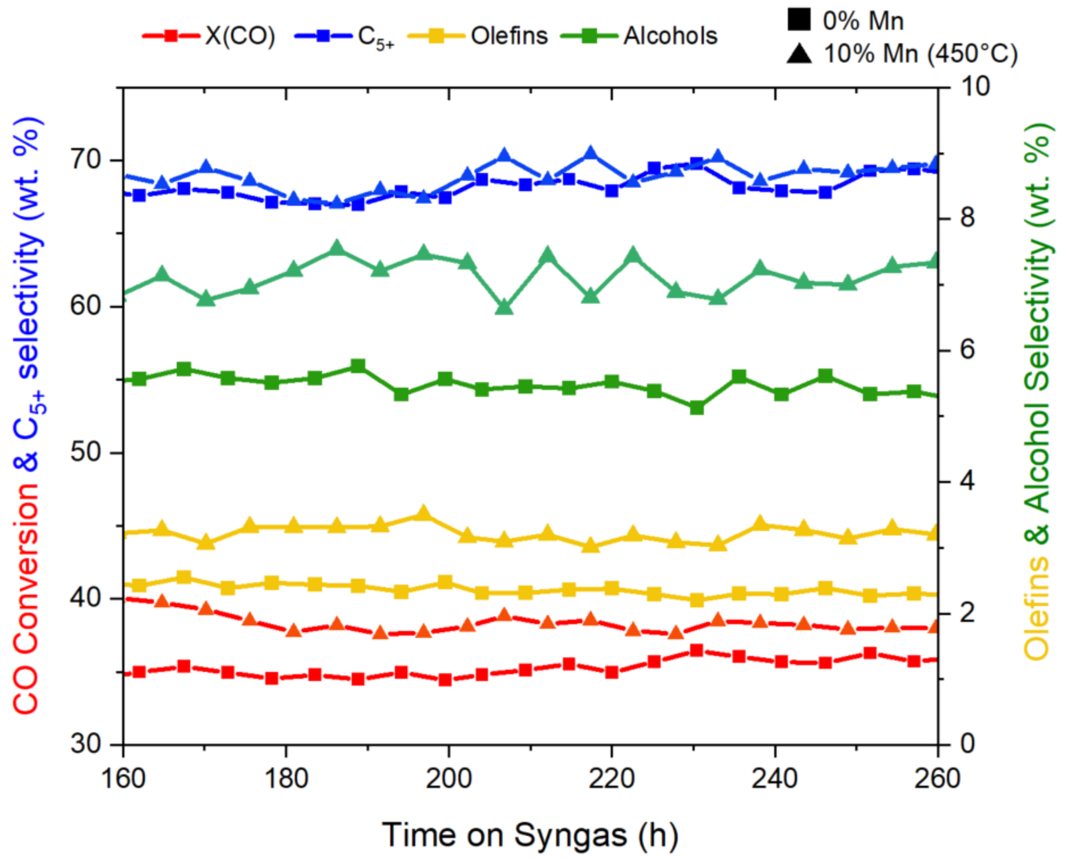

Figure S3 - CO conversion and selectivity to C5+, alcohols and olefins for the 0 % and 10 % Mn (450 °C) sample for the reaction time from 160 to 300 h at 30 barg and 210-240 °C.

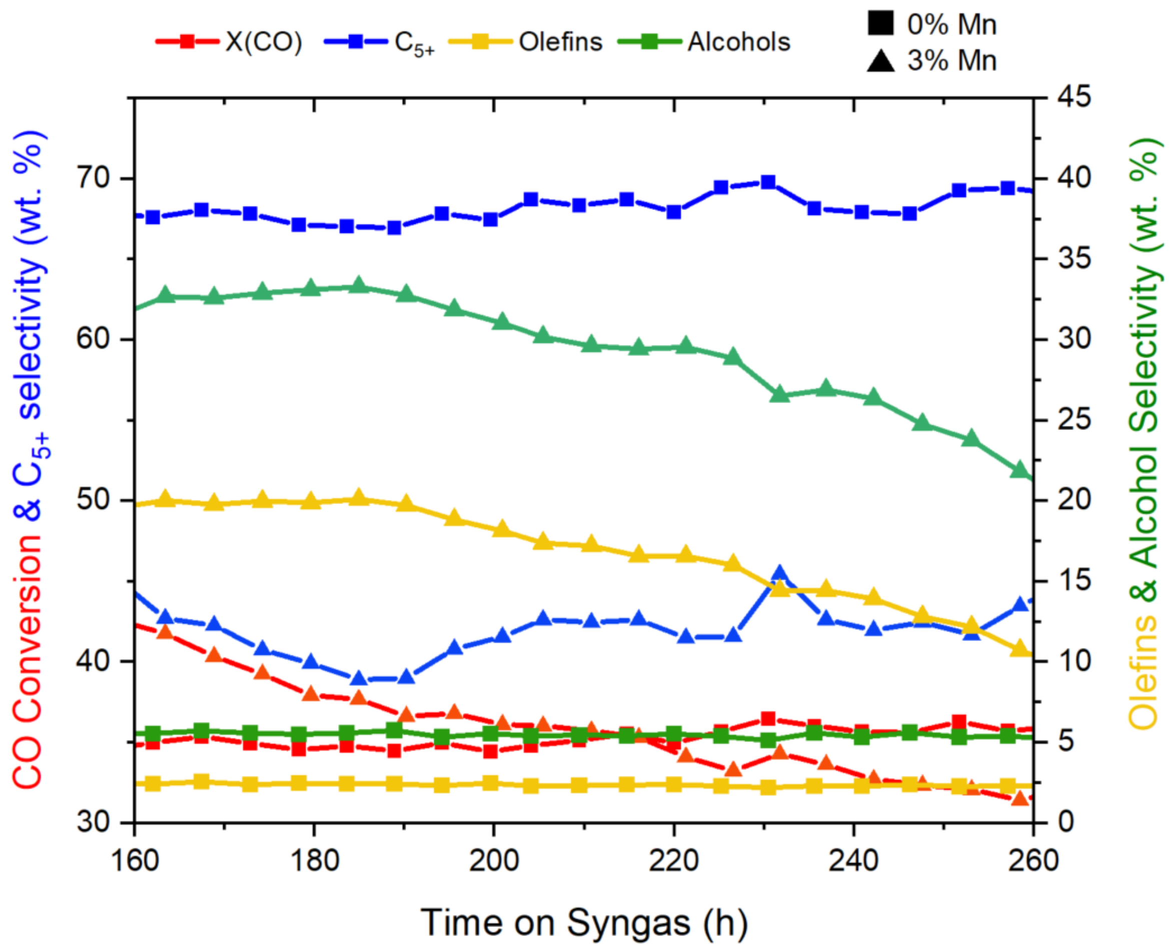

Figure S4 - CO conversion and selectivity to C5+, alcohols and olefins for the 0% and 3% Mn sample for the reaction time from 160 to 300 h at 30 barg and 210-240 °C.

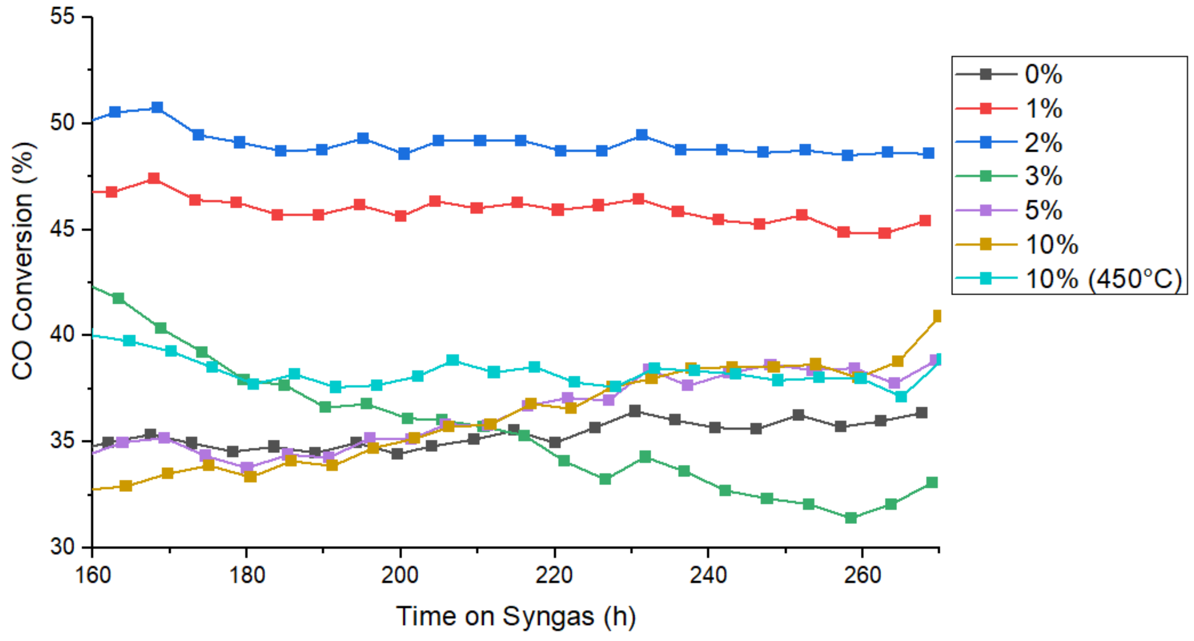

Figure S5 - CO conversion for all the samples tested from 160 – 270 h at 30 barg and 210-240 °C.

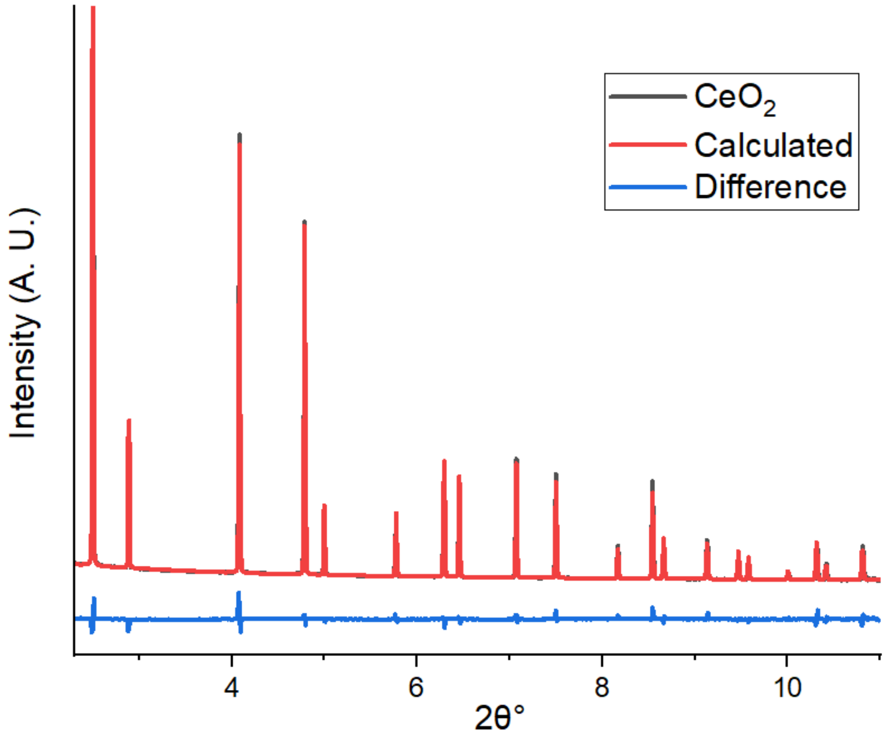

Figure S6 – XRD pattern of the CeO_2 calibrant, the calculated refined pattern and the difference pattern.

Table S2 - Rietveld Refinement results of the XRD-CT mean patterns illustrating the phase wt. %, CS (crystallite size in nm) and LP (lattice parameters) of the samples extracted after 150 h and 300 h. The Co_2C wt. % increases with Mn loading whilst the Co^0 metal phases (FCC and HCP) decrease with increasing Mn wt. % beyond 5 %. The 10 % samples reduced at 450 °C resulted in the production of Mn titanates ($MnTiO_3$).

[a] Reduction at 450 °C

**Phase**		**Mn wt. %**							
**Time (h)**		**150**	**300**						
		**5 %**	**0 %**	**1 %**	**2 %**	**3 %**	**5 %**	**10 %**	**10 %[a]**
R_{wp} (%)		6.4	6.9	6.2	6.2	6.2	6.7	7.9	7.9
Co_2C	Wt. %	7.5	-	0.6	0.9	3.6	8.9	7.1	-
	CS	7.5	-	-	-	10.6	9.3	11.7	-
	LP (Å)	2.89	-	2.99	2.99	2.89	2.89	2.89	-
		4.45	-	4.61	4.65	4.45	4.45	4.45	-
		4.37	-	4.28	4.26	4.37	4.37	4.37	-
FCC	Wt. %	0.4	3.5	3.3	3.6	1.6	0.1	0.0	2.9
	CS	3.54	10.5	8.1	7.7	5.2	-	-	11.2
	LP (Å)	3.54	3.54	3.54	3.54	3.54	-	-	3.54
HCP	Wt. %	0.2	4.9	4.8	3.8	3.1	0.2	0.7	5.3
	CS	-	2.6	2.2	2.2	7.1	-	9.9	2.9
	LP (Å)	2.51	2.51	2.51	2.52	2.51	2.51	2.50	2.51
		4.10	4.10	4.10	4.06	4.08	4.01	4.21	4.08
CoO	Wt. %	0.9	0.3	0.1	0.2	0.5	1.2	3.1	0.4
	CS	4.0	7.1	3.0	3.7	-	3.3	2.6	5.7

	LP (Å)	4.22	4.28	4.26	4.27	4.24	4.24	4.29	4.28
Rutile	Wt%	10.8	13.5	12.8	11.4	11.4	10.8	8.8	2.6
	CS	25.8	42.6	40.8	39.6	38.2	37.4	28.6	29.1
	LP (Å)	4.60	4.59	4.59	4.59	4.59	4.59	4.60	4.60
		2.96	2.95	2.96	2.96	2.96	2.96	2.96	2.96
Anatase	Wt. %	80.2	77.8	78.4	80.1	79.7	78.9	80.3	65.1
	CS	16.9	21.4	20.7	19.8	19.5	18.9	13.7	15.1
	LP (Å)	3.79	3.79	3.79	3.79	3.79	3.79	3.79	3.79
		9.51	9.50	9.51	9.50	9.50	9.50	9.50	9.50
$MnTiO_3$	Wt. %								23.7
	CS								20.8
	LP (Å)								5.14
									14.26

Table S3 –Errors from the Rietveld Refinement results of the XRD-CT mean patterns for the refined phase wt. %, CS (crystallite size in nm) and LP (lattice parameters) of the samples extracted after 150 h and 300 h.

[a] Reduction at 450 °C

**Phase**		**Mn wt. %**							
**Time (h)**		**150**	**300**						
		**5 %**	**0 %**	**1 %**	**2 %**	**3 %**	**5 %**	**10 %**	**10 %[a]**
R_{wp} (%)		6.4	6.9	6.2	6.2	6.2	6.7	7.9	7.9
Co_2C	Wt. %	0.696	-	0.078	0.108	0.097	0.139	0.161	-
	CS	0.603	-	-	-	1.36	0.63	1.12	
	LP (Å)	0.001	-	0.007	0.011	0.001	0.001	0.001	-
		0.045	-	0.014	0.022	0.002	0.001	0.002	-
		0.002	-	0.013	0.019	0.003	0.002	0.002	-
FCC	Wt. %	0.035	0.080	0.074	0.079	0.087	0.047	0.083	0.087
	CS	1.11	1.34	0.73	0.66	1.95	-	-	1.34
	LP (Å)	0.006	0.001	0.001	0.001	0.003	-	-	0.001
HCP	Wt. %	0.036	0.138	0.128	0.114	0.097	0.052	0.089	0.177
	CS	-	0.27	0.21	0.25	1.25	-	5.7	2.84
	LP (Å)	0.008	0.003	0.003	0.004	0.001	0.003	0.007	0.003
		0.024	0.003	0.010	0.016	0.004	0.008	0.027	0.010
CoO	Wt. %	0.177	0.201	0.126	0.109	0.140	0.200	0.267	0.098
	CS	1.38	4.47	1.95	2.76	-	1.86	1.32	2.90
	LP (Å)	0.023	0.009	0.018	0.047	0.030	0.014	0.007	0.043

Rutile	Wt%	0.775	0.150	0.132	0.133	0.137	0.157	0.221	0.120
	CS	5.02	4.07	3.94	4.01	4.02	4.97	5.16	10.4
	LP (Å)	4e-4	3e-4	2e-4	3e-4	3e-4	4e-4	8e-4	0.001
		5e-4	3e-4	3e-4	3e-4	3e-4	4e-4	9e-4	0.002
Anatase	Wt. %	0.179	0.248	0.210	0.212	0.218	0.254	0.347	0.268
	CS	0.56	0.48	0.42	0.39	0.38	0.40	0.31	0.38
	LP (Å)	9e-5	1e-4	1e-4	1e-4	1e-4	1e-4	8e-4	2e-4
		4e-4	4e-4	4e-4	4e-4	4e-4	5e-4	9e-4	8e-4
$MnTiO_3$	Wt. %								0.219
	CS								0.83
	LP (Å)								3e-4
									0.002

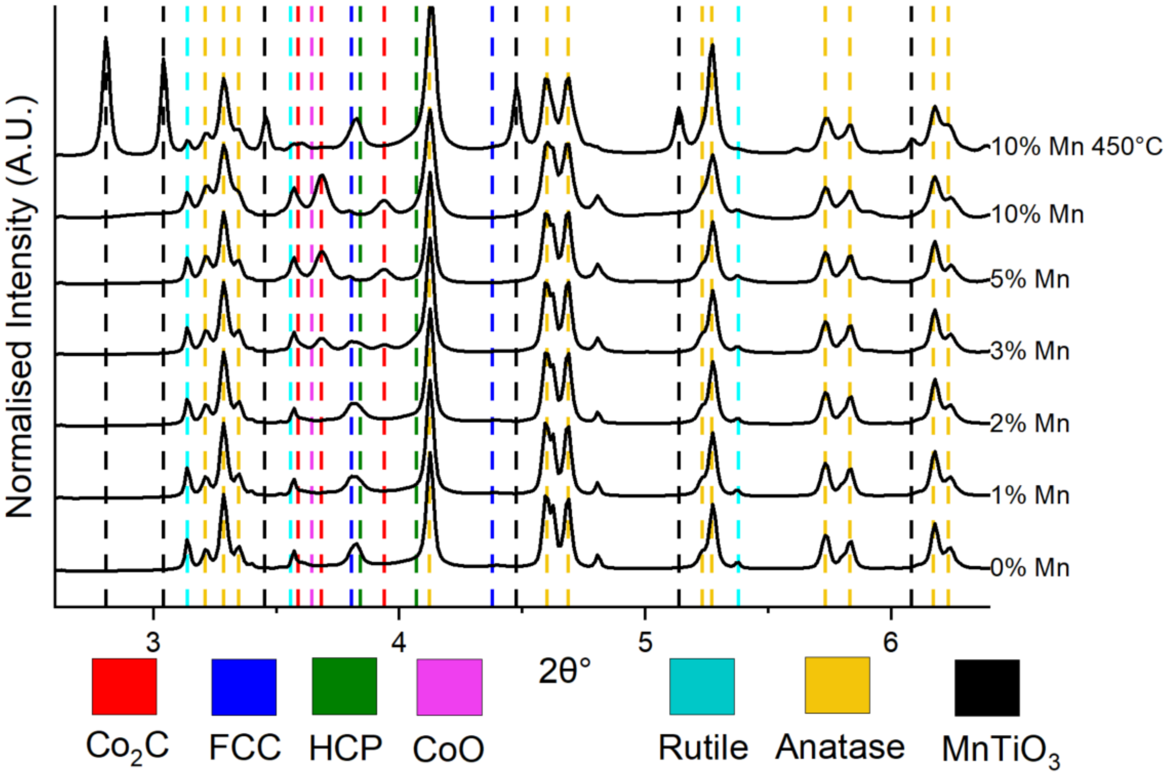

Figure S7 – Full mean XRD pattern of the samples with different Mn loading with indexing lines of all the phases present. FCC and HCP Co phases were present at low Mn loading (0-2 % Mn) and Co_2C was present at higher Mn loading (3-10 % Mn). A low percentage of $Co_x Mn_{1-x} O$ was present at higher Mn loadings (5-10 %) but its peaks were underneath the Co_2C peaks. Support phases, anatase and rutile, were present in all samples and $MnTiO_3$ was present in the sample reduced at 450 °C.

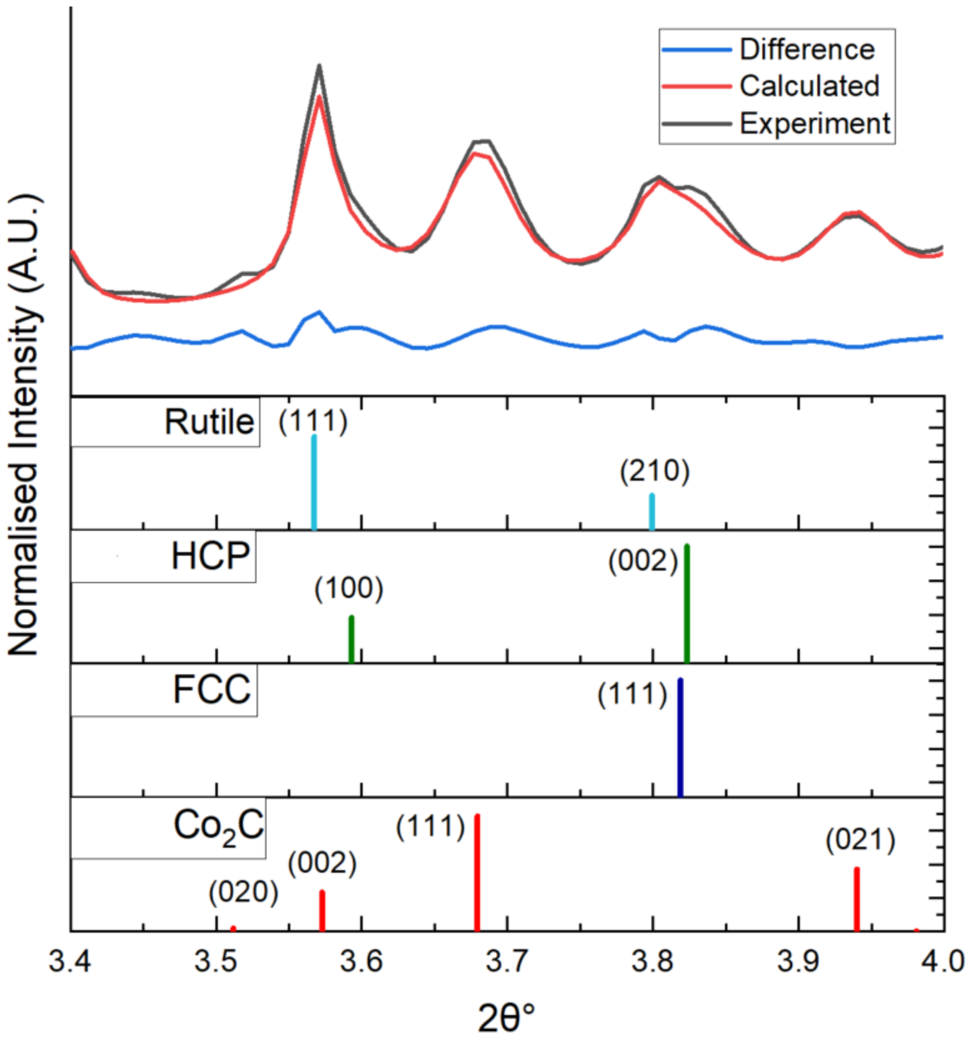

Figure S8 - Mean pattern refinement of the 3 % Mn reacted sample highlighting the 2θ° region with reflections from the different Co containing phases (Co_2C, FCC and HCP cobalt metal phases respectively). The experimental, refined and difference XRD patterns are displayed as well as the indexing lines for the reference patterns below. The peak shapes are not fitted exactly due to the presence of stacking faults in the FCC/HCP phase, but the R_{wp} is still low at 6.2 %.

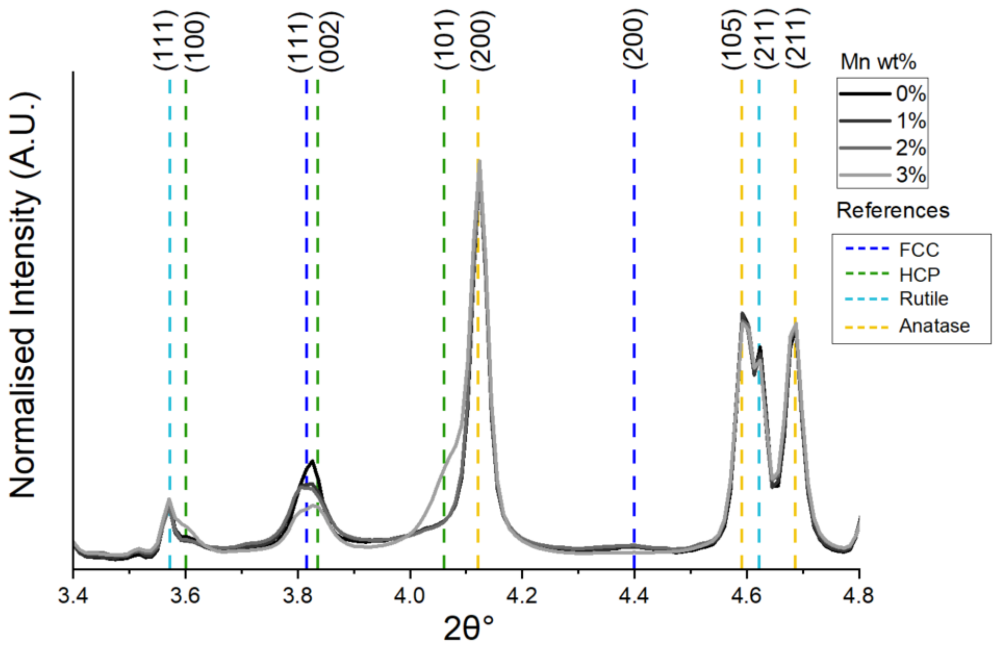

Figure S9 – XRD patterns of the 0-3 % Mn samples from the centre of the extrudates after 300 h of reaction. The 3 % Mn sample has more prominent HCP peaks at 3.60° and 4.07°.

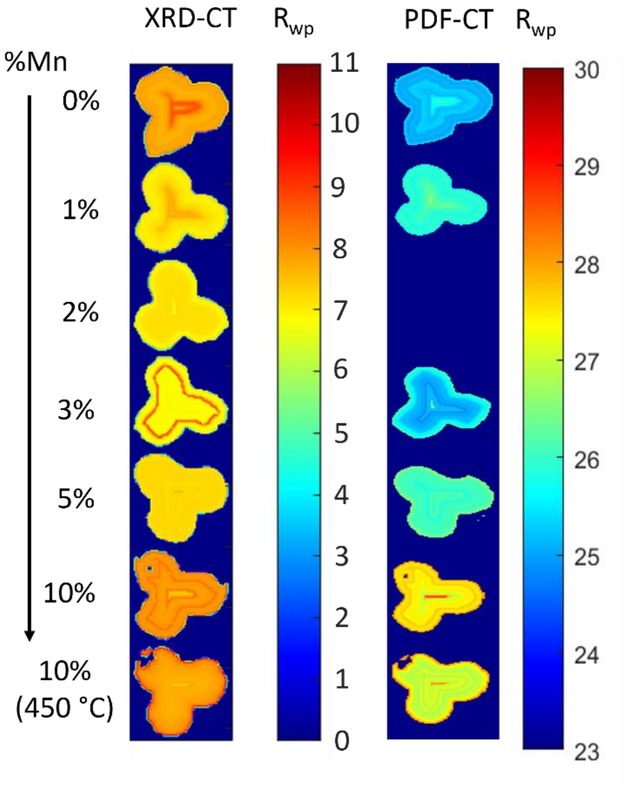

Figure S10 – R_{wp} maps for the XRD-CT and PDF-CT refinements where the XRD-CT R_{wp} was between 8 - 10 % indicating a good fit for all the samples. Increasing Mn loading resulted in a larger R_{wp} indicating increased disorder. The PDF-CT R_{wp} was between 23 - 28 % which indicates a tolerable fit for PDF data.

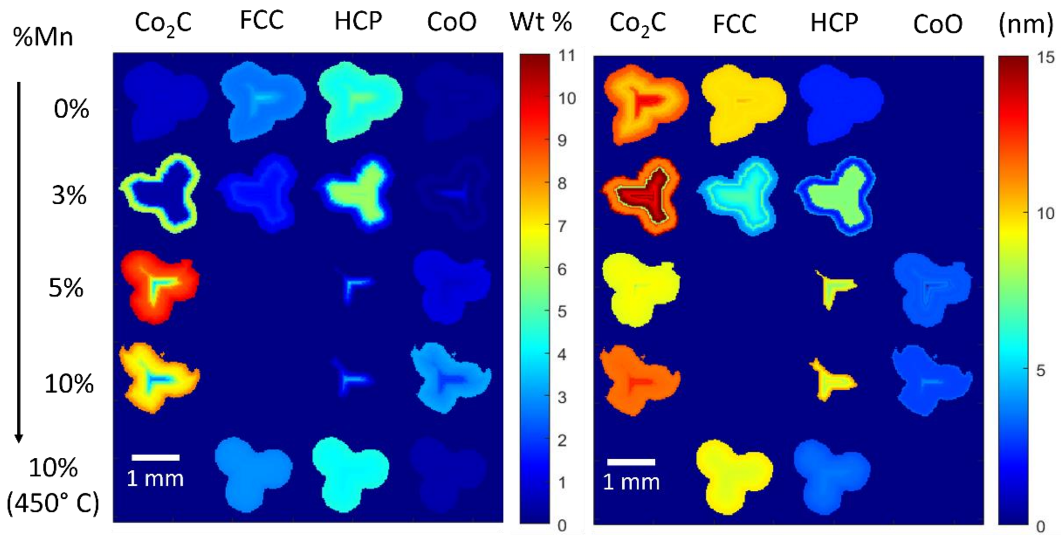

Figure S11 – Reconstructed XRD-CT 2D images of the catalytic pellets (extracted after 300 h) at alternative heights illustrating the refined wt. % percentage (left) and crystallite size (right) of the different phases present. The results at different heights show the same increase in Co_2C with Mn loading and egg-shell distribution with FCC/HCP in the centre and carbide on the periphery of the pellets.

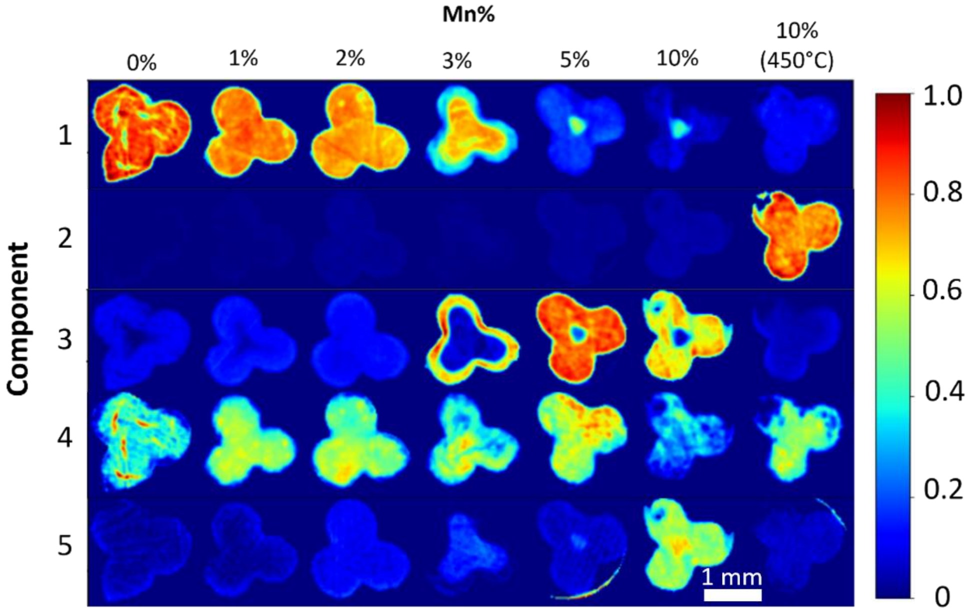

Figure S12 – XRD NMF-generated masks highlighting the spatial intensity variations of the 5 components within the catalytic samples. Component 1 exhibited similarities to the FCC/HCP phases, which tended to concentrate in the core of the catalyst at higher Mn loadings, while Component 3 contained the Co_2C phase which was found on the catalyst periphery.

Table S4 – XRD Rietveld refinement results from refining the NMF-generated components. Component 1 was found to contain FCC/HCP phases whilst component 2 contained the $MnTiO_3$. Component 3 contained the Co_2C phase whilst components 4 and 5 had a mixture of different phases. % int. refers to the percentage intensity of each component.

	% int.	Parameter	Co_2C	FCC	HCP	CoO	Rutile	Anatase	$MnTiO_3$	Wax (CH_2)
**1**	59	wt. %	-	4.1	3.1	-	10.1	64.9	-	14.7
		CS (nm)	-	5.5	4.9	-	43.4	20.6	-	36.7
		LP (Å)	-	3.54	2.51	-	4.59	3.79	-	2.55
			-	-	4.07	-	2.96	9.50	-	4.96
			-	-	-	-	-	-	-	7.45
**2**	16	wt. %	-	3.0	3.8	-	2.1	51.6	21.6	17.4
		CS (nm)	-	7.9	3.8	-	-	15.3	20.5	41.4
		LP (Å)	-	3.54	2.5	-	4.59	3.79	5.1	2.55
			-	-	4.1	-	2.96	9.50	14.3	4.96
			-	-	-	-	-	-	-	7.46
**3**	22	wt. %	7.2	-	-	-	8.6	65.4	-	18.3
		CS (nm)	9.6	-	-	-	37.1	8.6	-	40.6
		LP (Å)	2.89	-	-	-	4.59	3.8	-	2.55
			4.45	-	-	-	2.96	9.5	-	4.96
			4.37	-	-	-	-	-	-	7.44
**4**	2	wt. %	4.59	1.3	1.0	-	8.8	61.2	-	23.1
		CS (nm)	9.7	6.0	5.7	-	41.2	19.8	-	45.9
		LP (Å)	2.9	3.54	2.50	-	4.59	3.79	-	2.55
			4.4	-	4.08	-	2.96	9.50	-	4.96
			4.4	-		-	-	-	-	7.44
**5**	0.3	wt. %	1.4	-	4.75	2.75	5.67	60.6	-	24.8
		CS (nm)	13.0	-	5.7	2.3	-	14.5	-	34.9
		LP (Å)	2.91	-	2.50	4.28	4.59	3.79	-	2.55
			4.44	-	4.09	4.28	2.96	9.49	-	4.96
			4.36	-	4.75	-	-	-	-	7.44

Table S5 – Errors from the XRD Rietveld refinement of the NMF-generated components.

	% int.	Parameter	Co_2C	FCC	HCP	CoO	Rutile	Anatase	$MnTiO_3$	Wax (CH_2)
**1**	59	wt. %	-	0.12	0.14	-	0.18	0.40	-	0.30
		CS (nm)	-	0.70	1.43	-	8.90	1.45	-	3.20
		LP (Å)	-	1e-3	2e-3	-	5e-4	2e-4	-	1e-3
			-	-	7e-3	-	5e-4	7e-4	-	1e-3
			-	-	-	-	-	-	-	2e-3
**2**	16	wt. %	-	0.10	0.16	-	0.11	0.31	0.23	0.31
		CS (nm)	-	1.33	0.85	-	-	0.96	3.1	19.7
		LP (Å)	-	3e-3	3e-3	-	2e-3	3e-4	6e-4	2e-3
			-	-	0.01	-	3e-3	1e-3	3e-3	2e-3
			-	-	-	-	-	-	-	3e-3
**3**	22	wt. %	0.15	-	-	-	0.19	0.35	-	0.34
		CS (nm)	1.98	-	-	-	9.16	1.23	-	5.61
		LP (Å)	1e-3	-	-	-	6e-4	2e-4	-	1e-3
			2e-3	-	-	-	6e-4	6e-4	-	1e-3

			2e-3	-	-	-	-	-	-	2e-3
**4**	2	wt. %	0.13	0.10	0.13	-	0.19	0.36	-	0.36
		CS (nm)	2.10	2.09	3.06	-	10.4	1.51	-	9.7
		LP (Å)	2e-3	4e-3	7e-3	-	5e-4	2e-4	-	8e-4
			3e-3	-	0.02	-	6e-4	6e-4	-	1e-3
			3e-3	-	-	-	-	-	-	1e-3
**5**	0.3	wt. %	0.19	-	0.25	0.48	0.29	0.66	-	0.64
		CS (nm)	9.02	-	1.36	5.32	-	0.68	-	23.8
		LP (Å)	0.01	-	4e-3	0.12	2e-3	3e-4	-	3e-3
			0.02	-	0.014	0.12	2e-3	1e-3	-	2e-3
			0.03	-	-	-	-	-	-	4e-3

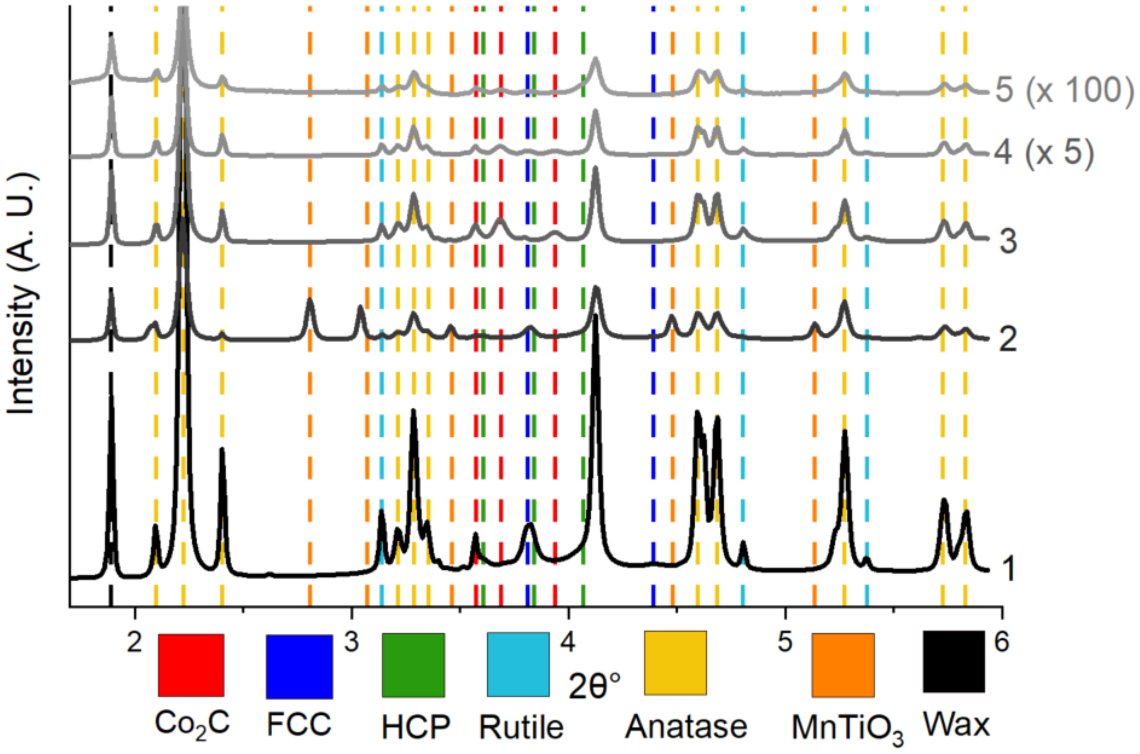

Figure S13 – XRD patterns of the NMF-generated components. All components contained the support phases as the support was homogeneous. Component 1 contained predominantly FCC/HCP Co, component 3 contained Co_2C and component 2 contained $MnTiO_3$. Components 4 and 5 were multiplied by 5 and 100, respectively, for visibility.

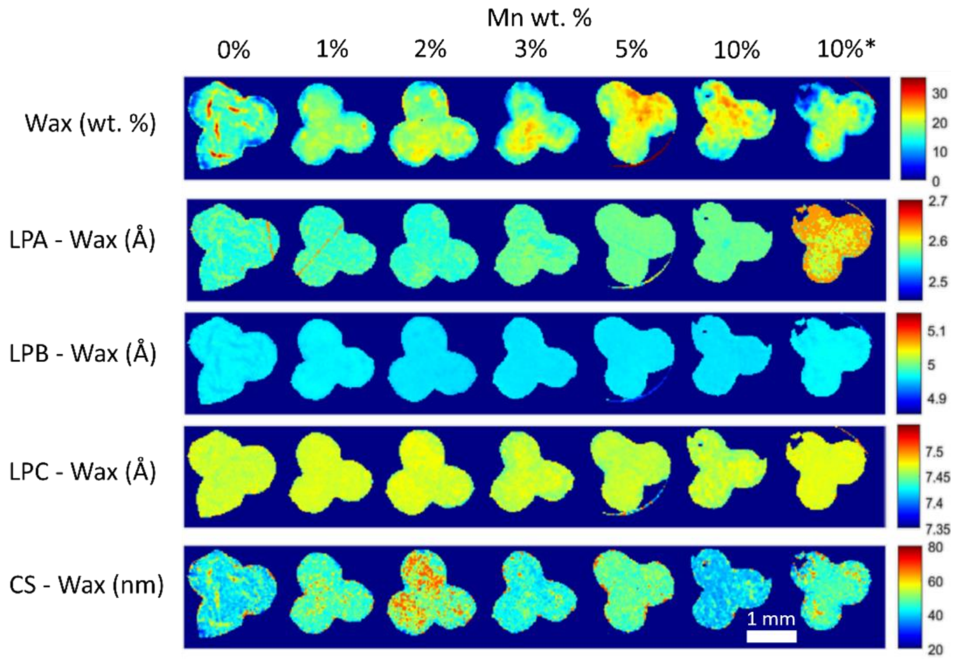

*Figure S14 - XRD-CT maps of the refined parameters of the wax content (wt. %), lattice parameters (LPA, LPB and LPC), and crystallite size (CS). *Reduced at 450 °C during preparation.*

Table S6 – Real-space Refinement results of the PDF-CT mean patterns illustrating the phase wt. %, CS (crystallite size) and LP (lattice parameters). The results correlate well with the XRD refinement. Co_2C wt. % increases with Mn loading whilst the Co^0 metal phases (FCC and HCP) decrease with increasing Mn wt. % beyond 5 %.

[a] Reduction at 450° C

**Phase**	**Parameter**	**Mn wt. %**					
		**0 %**	**1 %**	**3 %**	**5 %**	**10 %**	**10 %[a]**
	R_{wp}(%)	21.6	21.5	21.1	22.1	23.5	25.8
Co_2C	Wt. %	1.1	1.1	3.7	9.1	9.5	1.3
	CS (nm)	-	-	-	15.6	12.0	-
	LP (Å)	2.87	2.87	2.88	2.88	2.88	2.87
		4.41	4.42	4.43	4.43	4.43	4.42
		4.39	4.39	4.37	4.37	4.37	4.39
FCC	Wt. %	5.7	5.4	1.7	-	0.0	5.0
	CS (nm)	4.0	3.7	4.1	-	-	8.5
	LP (Å)	3.53	3.54	3.54	-	-	3.53
HCP	Wt. %	5.0	4.8	5.4	0.3	0.2	2.2
	CS (nm)	3.9	3.6	4.2	-	-	5.6
	LP (Å)	2.49	2.50	2.50	2.58	2.60	2.73
		4.09	4.09	4.07	4.04	4.05	3.96
CoO	Wt. %	1.1	1.1	1.0	1.1	5.2	1.7

	CS (nm)	2.1	1.9	1.6	1.1	1.0	2.6
	LP (Å)	4.25	4.26	4.26	4.26	4.34	4.29
Rutile	Wt. %	15.8	15.2	13.9	12.8	11.6	5.2
	CS (nm)	4.3	4.4	4.4	4.6	4.2	4.6
	LP (Å)	4.59	4.59	4.59	4.59	4.60	4.62
		2.95	2.95	2.95	2.95	2.94	2.91
Anatase	Wt. %	71.1	72.1	74.0	76.2	73.2	59.3
	CS (nm)	5.1	5.1	5.0	5.0	4.5	4.6
	LP (Å)	3.78	3.78	3.78	3.78	3.78	3.78
		9.47	9.47	9.47	9.47	9.47	9.47
$MnTiO_3$	Wt. %						25.8
	CS (nm)						9.30
	LP (Å)						5.12
							14.23

Table S7 – Errors from the Real-space Refinement results of the PDF-CT mean patterns for the phase wt. %, CS (crystallite size) and LP (lattice parameters).

[a] Reduction at 450° C

**Phase**	**Parameter**	**Mn wt. %**					
		**0 %**	**1 %**	**3 %**	**5 %**	**10 %**	**10 %[a]**
	R_{wp}(%)	21.6	21.5	21.1	22.1	23.5	25.8
Co_2C	Wt. %	0.204	0.205	0.214	0.281	0.252	0.103
	CS (nm)	-	-	-	3.96	0.46	-
	LP (Å)	0.006	0.006	0.002	0.001	0.001	0.005
		0.009	0.010	0.003	0.002	0.001	0.008
		0.010	0.011	0.003	0.002	0.001	0.009
FCC	Wt. %	0.157	0.166	0.160	0.17	0.16	0.19
	CS (nm)	0.15	0.14	0.51	0.01	-	0.42
	LP (Å)	6e-4	7e-4	0.003	-	0.74	7e-4
HCP	Wt. %	0.338	0.343	0.314	0.192	0.178	0.239
	CS (nm)	0.30	0.33	0.41	-	-	0.084
	LP (Å)	0.002	0.003	0.002	0.004	0.006	0.001
		0.007	0.008	0.006	0.014	0.020	0.004
CoO	Wt. %	0.221	0.012	0.200	0.185	0.515	0.316

	CS (nm)	0.32	0.42	0.47	0.69	0.21	0.23
	LP (Å)	0.009	0.005	0.005	0.004	0.005	0.005
Rutile	Wt. %	0.317	0.313	0.160	0.306	0.321	0.28
	CS (nm)	0.13	0.15	0.16	0.20	0.19	0.19
	LP (Å)	9e-4	9e-4	9e-4	0.001	0.001	0.002
		0.001	0.001	0.001	0.001	8e-4	0.003
Anatase	Wt. %	0.434	0.418	0.441	0.423	0.518	0.369
	CS (nm)	0.043	0.042	0.039	0.039	0.035	0.401
	LP (Å)	2e-4	2e-4	2e-4	2e-4	2e-4	2e-4
		7e-4	7e-4	7e-4	7e-4	8e-4	8e-4
$MnTiO_3$	Wt. %						0.307
	CS (nm)						0.50
	LP (Å)						6e-4
							0.002

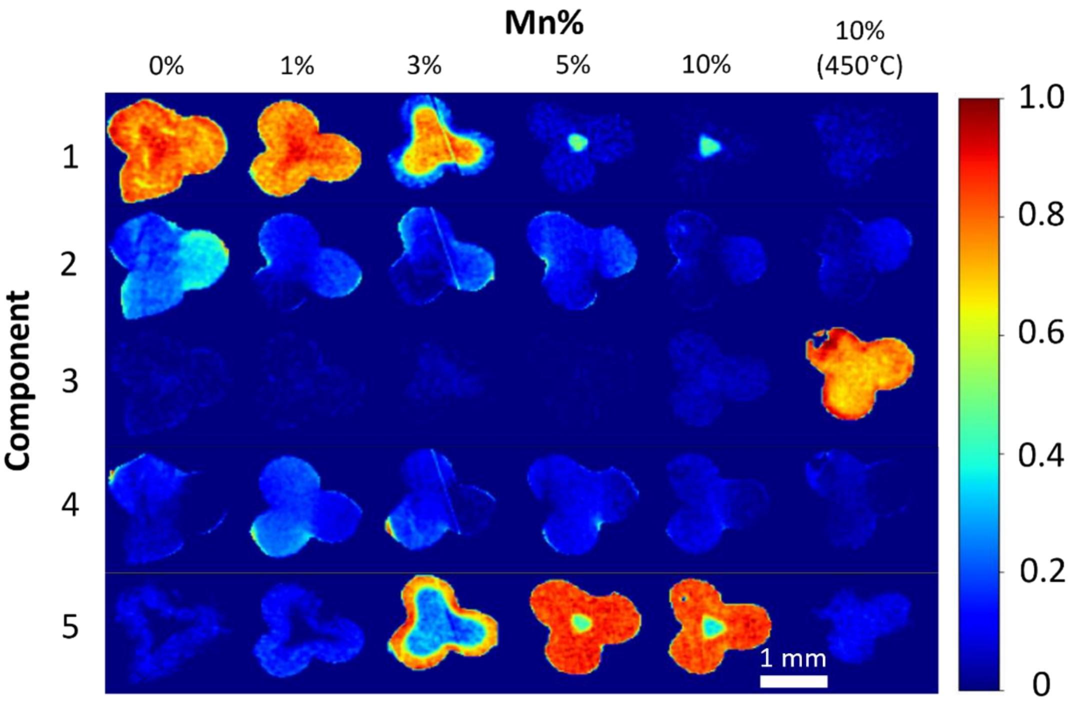

Figure S15 – PDF-CT NMF-generated masks highlighting the spatial intensity variations of the 5 components within the catalytic samples. Component 1 exhibited similarities to the FCC/HCP phases, which tended to concentrate in the core of the catalyst at higher Mn loadings, while Component 5 contained the Co_2C phase which was found on the catalyst periphery.

Table S8 – PDF Real-space refinement results from refining the NMF-generated components. Component 1 was found to contain FCC/HCP phases whilst component 3 contained the $MnTiO_3$. Component 5 contained the Co_2C phase whilst components 2 and 4 had a mixture of different phases but had low intensity. % int. refers to the percentage intensity of each component.

	%. int	Parameter	Co_2C	FCC	HCP	CoO	Rutile	Anatase	$MnTiO_3$
**1**	43	wt. %	-	5.4	5.7	4.7	14.5	69.7	-
		CS (nm)	-	3.4	4.1	0.8	4.5	5.0	-
		LP (Å)	-	3.53	2.49	4.44	4.59	3.78	-
			-	-	4.10	-	2.95	9.47	-
			-	-	-	-	-	-	-
**2**	0.1	wt. %	-	5.0	4.1	4.4	13.1	67.4	-
		CS (nm)	-	5.3	3.8	0.7	4.9	5.7	-
		LP (Å)	-	3.53	2.48	4.42	4.57	3.77	-
			-	-	4.10	-	2.4	9.44	-
			-	-	-	-	-	-	-
**3**	15	wt. %	-	6.0	6.6	3.0	5.0	52.1	27.3
		CS (nm)	-	3.5	3.7	0.9	4.5	4.8	6.8
		LP (Å)	-	3.53	2.50	4.41	4.60	3.78	5.1
			-	-	4.10	-	2.93	9.47	14.2
			-	-	-	-	-	-	-
**4**	0.2	wt. %	5.5	1.6	3.4	7.7	11.0	70.8	-
		CS (nm)	12.5	8.8	5.8	0.7	3.7	6.2	-
		LP (Å)	2.90	3.55	2.58	4.5	4.6	3.79	-
			4.42	-	4.09	-	2.96	9.50	-

			4.40	-	-	-	-	-	-
		wt. %	10.6	-	-	5.1	12.1	72.1	-
		CS (nm)	7.8	-	-	0.8	4.5	4.8	-
**5**	42	LP (Å)	2.88	-	-	4.41	4.59	3.78	-
			4.42	-	-	-	2.94	9.47	-
			4.37	-	-	-	-	-	-

Table S9 – Errors from the PDF Real-space refinement results of the NMF-generated components.

	%. int	Parameter	Co_2C	FCC	HCP	CoO	Rutile	Anatase	$MnTiO_3$
		wt. %	-	0.12	0.24	0.37	0.22	0.42	-
		CS (nm)	-	0.09	0.28	0.68	0.12	0.03	-
**1**	43	LP (Å)	-	5e-4	2e-3	7e-3	6e-4	1e-4	-
			-	-	5e-3	-	8e-4	5e-4	-
			-	-	-	-	-	-	-
		wt. %	-	0.14	0.34	0.60	0.30	0.61	-
		CS (nm)	-	0.23	0.50	0.12	0.20	0.05	-
**2**	0.1	LP (Å)	-	7e-4	3e-3	0.01	1e-3	2e-4	-
			-	-	0.01	-	1e-3	7e-4	-
			-	-	-	-	-	-	-
		wt. %	-	0.08	0.18	0.23	0.15	0.26	0.31
		CS (nm)	-	0.73	0.20	0.09	0.27	0.03	0.16
**3**	15	LP (Å)	-	4e-4	1e-3	9e-3	2e-3	2e-4	5e-4
			-	-	4e-3	-	2e-3	6e-4	2e-3
			-	-	-	-	-	-	-
		wt. %	0.50	0.12	0.31	0.67	0.39	0.71	-
		CS (nm)	3.0	2.0	1.25	0.08	0.22	0.66	-
**4**	0.2	LP (Å)	2e-3	3e-3	5e-3	0.01	2e-3	2e-3	-
			3e-3	-	0.01	-	2e-3	8e-3	-
			4e-3	-	-	-	-	-	-
		wt. %	0.31	-	-	0.33	0.21	0.37	-
		CS (nm)	0.45	-	-	0.06	0.14	0.03	-
**5**	42	LP (Å)	7e-3	-	-	7e-3	8e-3	1e-4	-
			1e-3	-	-	-	1e-3	5e-4	-
			1e-3	-	-	-	-	-	-

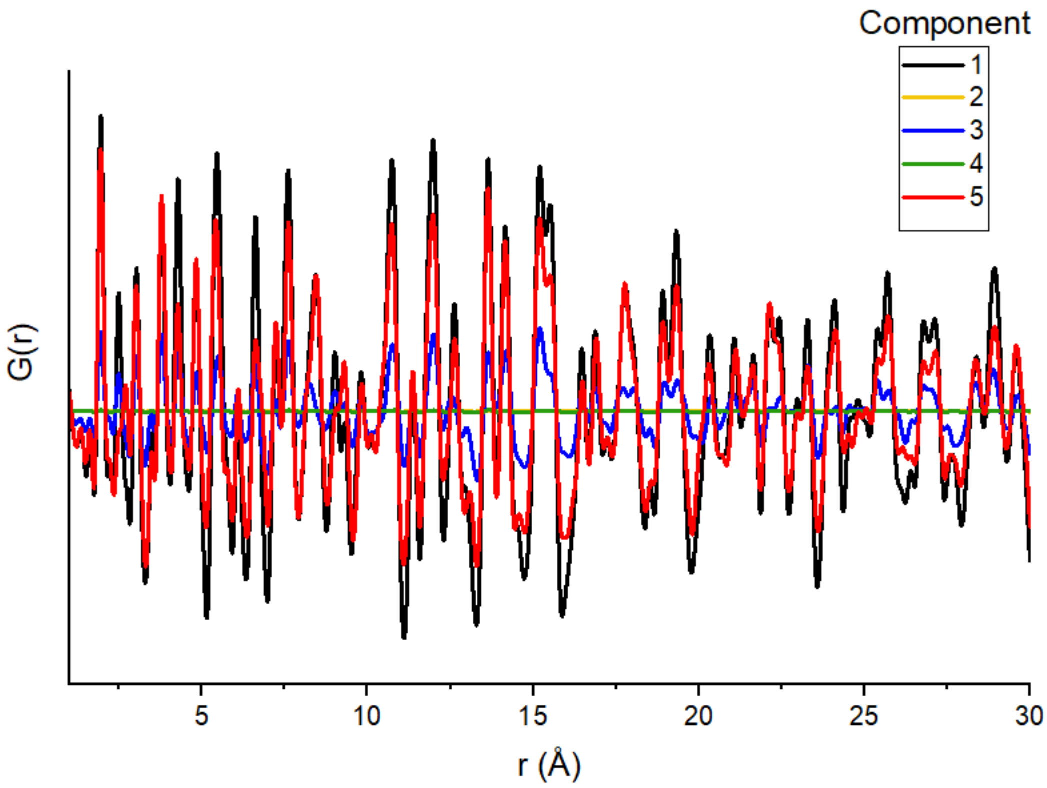

Figure S16 – PDF of the NMF-generated components. All components contained the support phases as the support was homogeneous. Component 1 contained predominantly FCC/HCP Co, component 5 contained Co_2C and component 3 contained $MnTiO_3$.

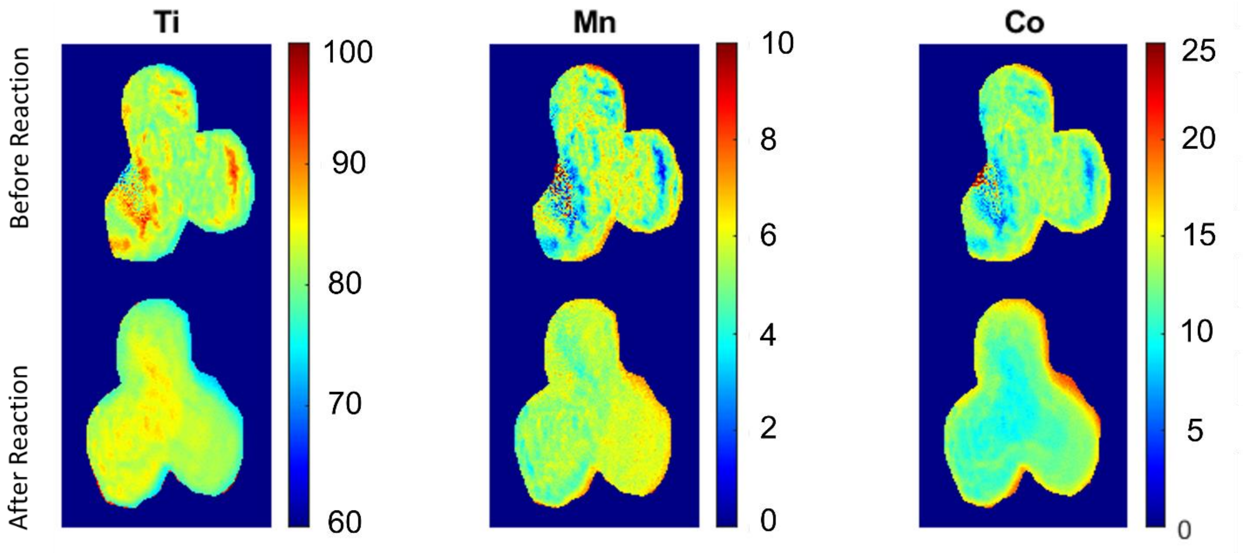

Figure S17 – µ-XRF images (intensity (%)) of the cross-sections (right) of the 3 wt. % Mn samples extracted before and after reaction for 300 h. The Mn and Co are found to be co-located.

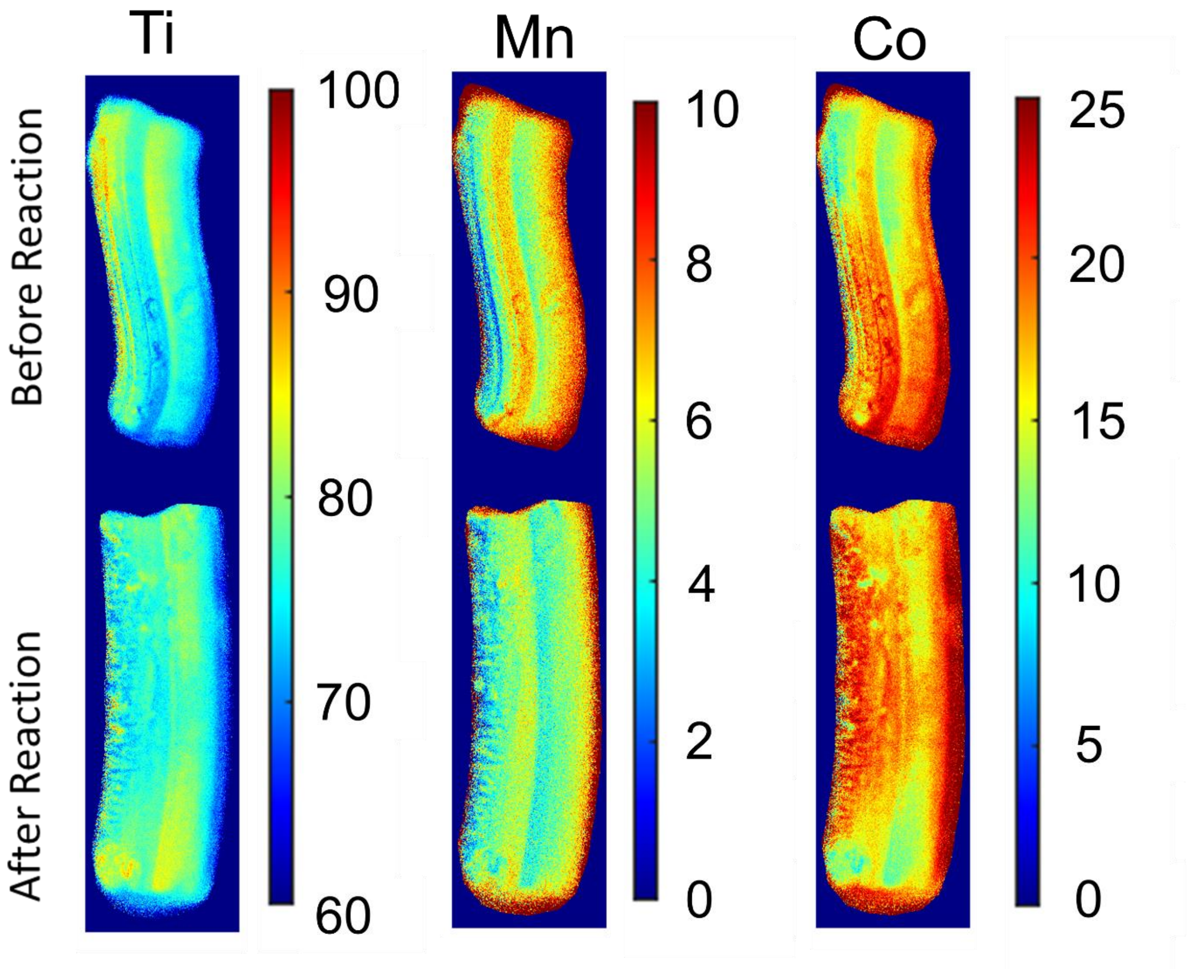

Figure S18 - µ-XRF images (intensity (%)) of the lateral faces of the 3 wt. % Mn samples extracted before and after reaction for 300 h.

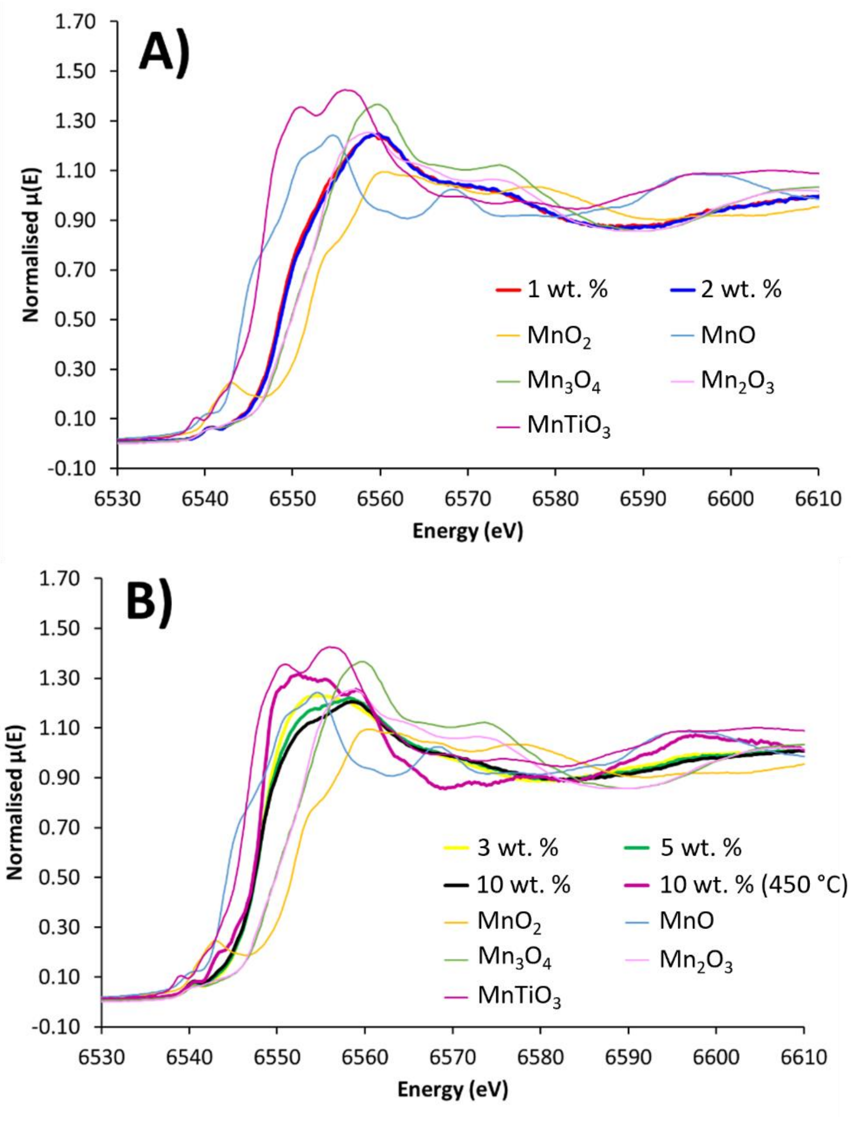

Figure S19 - Comparing Mn K-edge spectra of A) 1 and 2 wt. % CoMn/TiO_2 species and B) 3, 5, 10 and 10 wt. % Used CoMn/TiO_2 samples with a range of oxidic standards.
